# Review on Cathode Stabilization by Electrolyte Engineering in Aqueous Batteries

**DOI:** 10.1007/s40820-025-02048-w

**Published:** 2026-01-28

**Authors:** Ronggen Zhang, Xu Liu, Na Gao, Dandan Yin, Xingwang Chen, Hongyang Zhao, Shujiang Ding

**Affiliations:** School of Chemistry, Engineering Research Center of Energy Storage Materials and Devices, Ministry of Education, Xi’an Jiaotong University, Xi’an, 710049 People’s Republic of China

**Keywords:** Aqueous battery, Cathode materials, Electrolyte modification

## Abstract

The stability of cathode materials is a crucial factor that influence the overall performance of aqueous batteries. Electrolyte greatly influences on the stability of cathode material due to the complexed electrochemical–chemical reactions at the interfaces. Therefore, electrolyte engineering is a direct and powerful way to solve various problems at aqueous electrolyte interfaces. In this review article, we firstly summarized the fading mechanisms of different kinds of state-of-the-art aqueous battery cathodes including manganese/vanadium-based material, chalcogen and halogen materials, Prussian blue analogues, and Ni(OH)_2_ cathodes. Afterward, we reviewed recent progresses on electrolyte engineering on the stability of cathode materials such as bulk electrolyte modification, electrolyte additives, water-in-salt electrolytes, and hydrogel electrolytes. Finally, we proposed the issues that should be concerned in future electrolyte design for highly state aqueous battery cathodes.

## Highlights

- The fading mechanisms of different kinds of state-of-the-art aqueous battery cathodes including manganese/vanadium-based material, chalcogen and halogen materials, Prussian blue analogues, as well as Ni(OH)_2_ cathodes were summarized.
- Recent progresses on electrolyte engineering on the stability of cathode materials such as bulk electrolyte modification, electrolyte additives, water-in-salt electrolytes, and hydrogel electrolytes were systematically reviewed.
- The issues that should be concerned in future electrolyte design for highly state aqueous battery cathodes were proposed.

## Graphical abstract

**Figure:**
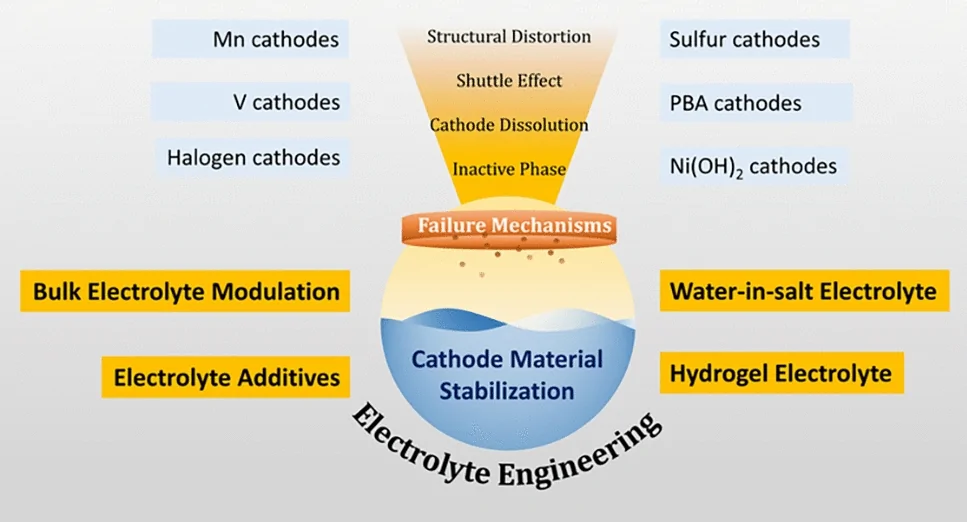

## Introduction

Electrochemical energy storage covers various aspects from the portable electronics power sources to the large-scale electricity storage. The safety issue has drawn considerable concerns due to the explosion and fire runaway caused by the flammable ester and ether-based electrolytes. Therefore, batteries based on aqueous electrolyte have been re-emphasized in the recent decade due to its irreplaceability in the energy storage condition that requires extreme safety. For instance, lead-acid batteries are more encouraged to be used in two-wheel electric vehicles by the Chinese government to reduce the fire accident caused by traditional lithium-ion batteries (LIBs). Aqueous nickel–zinc batteries are also more favorable to be used as uninterruptable power source (UPS) systems in computing power center than LIBs due to high safety requirement. However, the traditional lead-acid batteries and nickel-based alkaline batteries neither have low energy density or high levelized cost of energy (LCOE), which cannot fulfill the electrical energy storage needs in future growing high safety energy storage market. Therefore, novel aqueous batteries with higher energy density, longer cycle stability, and lower cost are proposed to meet these requirements.

Although batteries based on aqueous electrolyte have been developed for over a hundred years, with the rapid growing of LIBs and sodium-ion batteries (SIBs), aqueous batteries are not mentioned as much as these non-aqueous batteries in past decades and usually considered as “novel” electrochemical energy storage devices. This is due to the effort of researchers who tried to overcome the disadvantages of traditional aqueous batteries by developing novel battery systems other than lead-acid and alkaline nickel batteries, including aqueous LIBs, aqueous SIBs, and aqueous zinc-ion batteries. The most significant difference between traditional aqueous batteries and novel aqueous batteries is the pH of the electrolytes. For lead-acid and alkaline nickel-alkaline batteries, the electrolytes are either strong acidic or strong basic; meanwhile, for the newly developed aqueous batteries, the electrolytes are mostly pH neutral. The change of electrolyte pH greatly expanded the electrode material types. The change to a near-neutral pH environment reduces the corrosive effects associated with strong acids or bases, allowing sensitive electrode materials such as certain metal oxides and organic compounds to be stably used. In this way, the neutral electrolyte not only broadens the range of compatible electrode materials but also mitigates degradation pathways that limit the performance and cycle life of traditional aqueous batteries. Up to now, numerous kinds of aqueous battery electrode material have been developed, including metal oxides/sulfides, halogens, chalcogens, as well as small organic molecules and coordination polymers.

These newly developed aqueous batteries such as aqueous zinc-ion batteries (AZIBs), and aqueous lithium-/sodium-ion batteries (ALIBs, ASIBs) have been researched for over a decade, and unfortunately, only a few of these batteries are commercialized nor showing high potential to be used in practice. This discrepancy between prosperous scientific research and poorly commercialization potential lies in the different interest of academic and industrial groups. For academic researchers, it is more favorable to develop new chemistries or materials to gain academic reputations, papers, and research funds. For industrial researchers, it is more desired to upgrade mature technologies with higher performance or lower cost. Despite these discrepancies, it has to be admitted that there are barely aqueous batteries in current research that can be readily converted to commercial products. One of the most important reasons for this situation is the stability of cathode materials. Electrolyte engineering approaches can bridge this gap by improving long-term cycle stability under practical mass loading conditions. By improving cycle life and suppressing material degradation under realistic operating conditions, these approaches not only advance fundamental understanding but also address critical barriers to the commercialization of aqueous batteries.

The poor stability of aqueous battery cathode materials significantly influences on the overall performance. For instance, the dissolution and shuttling of cathodic species not only result in continuous loss of active materials, the oxidative ions would directly react with anode materials, which lead to anode corrosion, and decrease in Coulombic efficiency. Besides, the structural degradation of cathode material also aggravates the dissolution, altering the charge–discharge path and leading to local pH fluctuation, which leads to electrolyte depletion and gas formation.

**Fig. 1:**
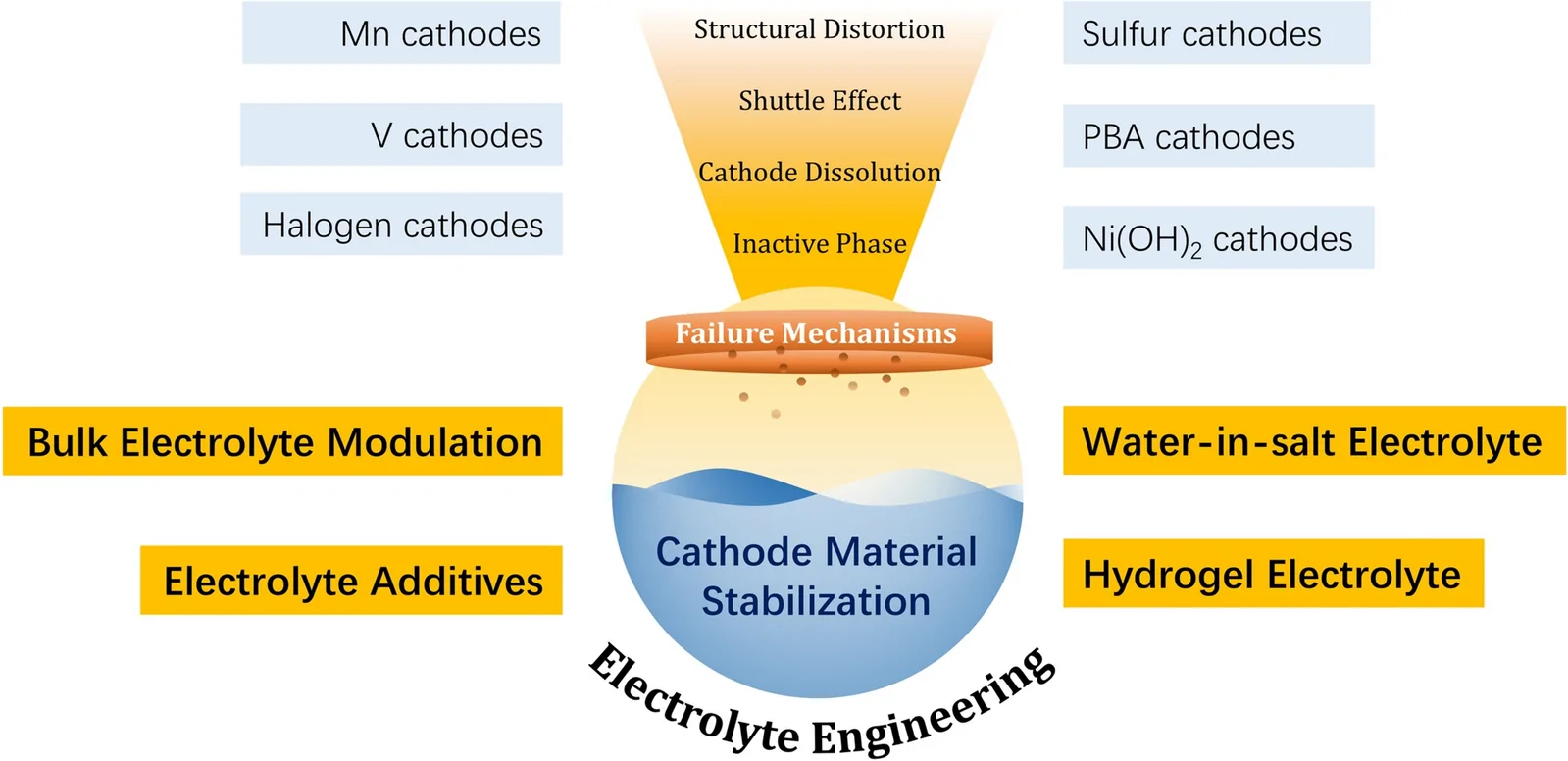
Schematic illustration of the review article concerning electrolyte engineering on stabilization of aqueous battery cathode materials

The degradation of aqueous battery cathode materials can be categorized into intrinsic degradation and extrinsic degradation. For intrinsic degradation, the cathode failure is caused by the irreversible structural deformation during charge–discharge process. Therefore, structural modifications such as element doping and phase modulation are effective to deal with intrinsic degradation. For extrinsic degradation, the cathode failure is caused by the attack of electrolyte components. Specifically, water molecules are highly reactive under cathode operation voltages. Water-induced degradation is the most significant problem in most of the aqueous batteries because water would hydrolyze the cathode materials, either by the dissociated hydroxide ions or protons. Therefore, reducing and tuning the water molecule activity is a highly effective way to enhance the stability of aqueous battery cathode materials, which needs delicate modification of the electrolyte (Fig. 1).

In this review article, we firstly discussed the failure mechanisms of different cathode materials. Mechanistic understandings are essential to precisely diagnose the fading of cathode materials, and therefore can we propose modification methods. In this section, we will firstly introduce the general failure mechanism of aqueous battery cathodes, after which the mechanism of dissolution and shuttling of cathodic species. Structural degradation induced by electrolyte is also discussed in this section. After mechanism discussion, we summarized recent progresses on the cathode stabilization strategies by electrolyte engineering, including mixed electrolytes, electrolyte additives, water-in-salt electrolytes, and hydrogel electrolytes. In the last section of this review article, we summarized the current electrolyte engineering methods, as well as proposing some perspective views on the future possible modification methods on aqueous electrolyte engineering.

## Cathode Failure in Aqueous Electrolyte

In aqueous battery, the degradation of cathode materials is a complicated issue. On the one hand, the cathode materials suffer from structural degradation by ion intercalation; on the other hand, the dissolution of cathode materials and the subsequent shuttle effect further aggravate the structural degradation of cathodes. These issues are prominent in cathodes based on redox-active transition metals and halogen species, such as Mn, Fe, halogens, and chalcogens compounds, which are inherently prone to partial solubilization in aqueous electrolytes during electrochemical cycling [1–3]. Cathode dissolution leads to irreversible loss of active materials, structural degradation, and rapid capacity fading. For instance, in manganese-based cathodes, Mn^2^⁺ ions readily dissolve into the electrolyte under mildly acidic or neutral conditions, undermining the structural integrity of the host lattice. Similarly, iodine-based cathodes suffer from the dissolution of polyiodide species, which can dynamically shuttle between electrodes.

The shuttle effect further exacerbates performance degradation by facilitating the uncontrolled migration of dissolved redox species, resulting in parasitic side reactions at the counter electrode. This leads to low Coulombic efficiency, increased self-discharge, and long-term instability [1]. The coexistence of dissolution and shuttle processes not only complicates the electrochemical environment but also introduces serious reliability concerns for practical aqueous battery deployment. These phenomena are not limited to a single class of materials but are rather intrinsic to the aqueous environment, where high ion mobility and solubility contribute to the universality and severity of these issues [4].

### Degradation of Manganese-Based Cathodes

**Fig. 2:**
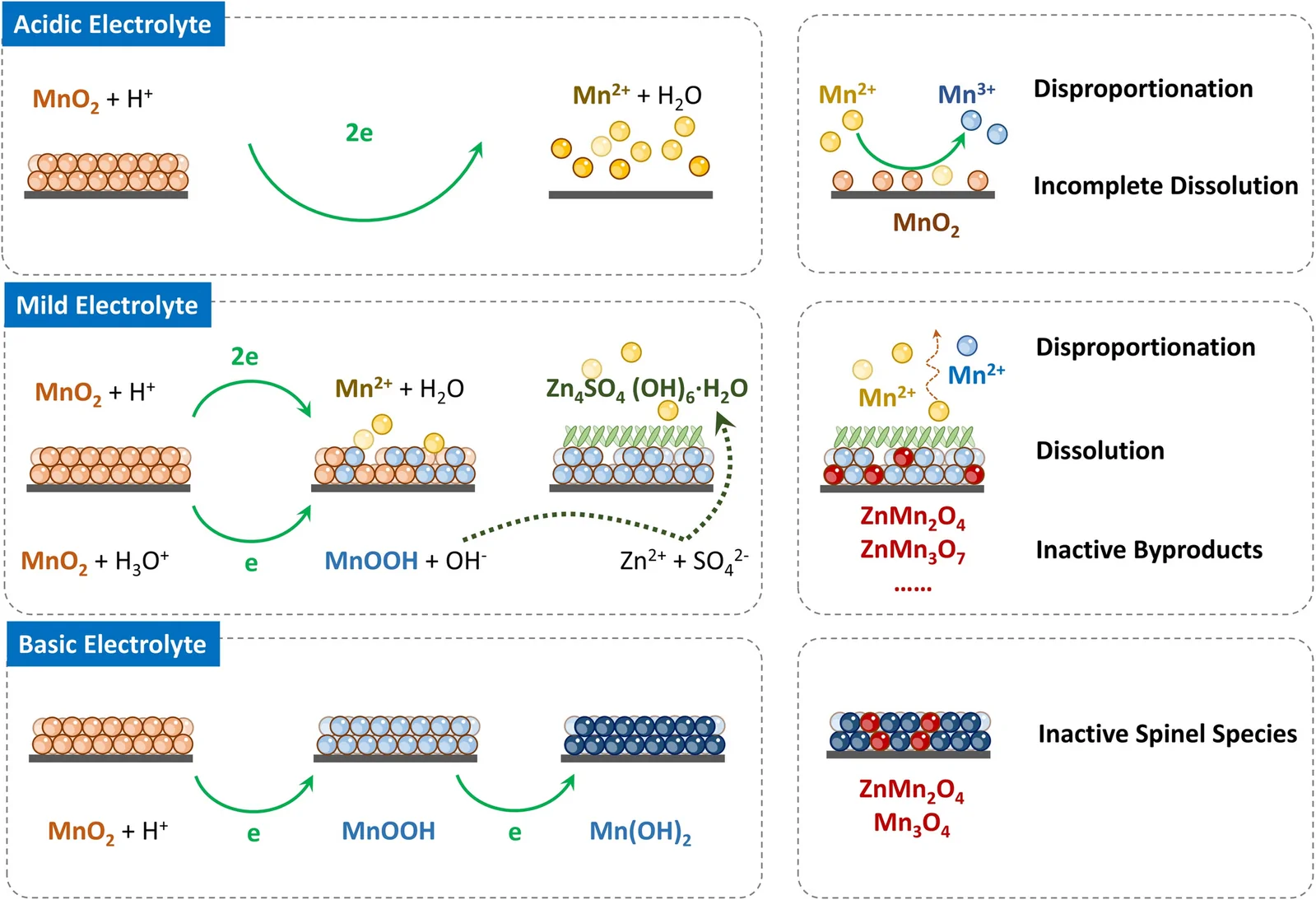
Degradation mechanism of manganese-based cathodes

Manganese oxide (MnO_2_) cathodes are one of the most studied cathodes for aqueous batteries due to its moderate operation voltage, large capacity, and low cost. However, MnO_2_ cathodes suffer from serious capacity fading during electrochemical cycles. The fading mechanisms were justified by various kinds of electrochemical and structural characterizations. To facilitate understanding, we respectively discuss the failure mechanisms of MnO_2_ cathodes under alkaline, neutral, and acidic pH conditions (Fig. 2).

Alkaline zinc–manganese batteries are among the most widely used power sources in electronic devices [1]. The typical cathode material is electrolytic manganese dioxide (EMD). The γ-MnO_2_ undergoes a two-electron structural transformation, eventually forming either α-MnOOH or γ-MnOOH [2–5]. This process involves a simultaneous Mn–O bond rearrangement and proton insertion. According to the Pourbaix diagram of manganese oxides, MnO_2_ undergoes a low-potential reaction (0.250 V vs. SHE) under high-pH conditions, potentially transforming into electrochemically inactive species. Upon coordination with protons, Mn^4+^ is reduced to Mn^3+^. During the second reduction step, Mn_3_O_4_ and Mn(OH)_2_ are formed. If zinc ions are present in the electrolyte, an electrochemically inactive compound, spinel-phase ZnMn_2_O_4_, may be produced via a subsequent reaction pathway.

1 $$2{\mathrm{MnOOH}} + {\mathrm{Zn}}\left( {{\mathrm{OH}}} \right)_{4}^{2 - } \to {\mathrm{ZnMn}}_{2} {\mathrm{O}}_{4} + 2{\mathrm{H}}_{2} {\mathrm{O}} + 2{\mathrm{OH}}^{ - }$$

The irreversibility of MnO_2_ is closely associated with the formation of spinel-phase Mn_3_O_4_ and ZnMn_2_O_4_ [6, 7]. The surfaces of cycled manganese oxide cathodes are typically covered with moss-like aggregates of electrochemically inactive by-products.

Mildly acidic or near-neutral electrolytes alleviate the drawbacks of alkaline systems, such as suppressing the formation of electrochemically inactive phases (e.g., ZnO on the anode and Mn_3_O_4_/ZnMn_2_O_4_ on the cathode) [11]. Nevertheless, new challenges including structural distortion of the MnO_2_ cathode and manganese dissolution arise in these media. The Jahn–Teller (J–T) distortion effect emerges as a predominant failure mechanism, particularly during Mn^3+^/Mn^4+^ conversion step in MnO_2_, where the asymmetric electronic configuration (t_2_g^3^eg^1^) induces characteristic octahedral distortion. This J–T distortion significant alters the lattice parameters, including notable variations in c/a axial ratios, which severely compromise ionic diffusion pathways, as evidenced by the substantial interlayer spacing fluctuations (7.0 Å) observed in δ-MnO_2_ structures. Phase transition-induced structural deformations constitute another major degradation pathway, where reversible phase transformations (e.g., P2-O2 transitions) during electrochemical cycling generate substantial volumetric changes ranging of 2%–15%. These dimensional instabilities mechanically destabilize the crystal framework through microcrack propagation and active material delamination. Furthermore, the high charge density of Zn^2+^ (0.74 Å ionic radius) induces pronounced polarization effects within the host matrix, creating strong electrostatic interactions with anionic frameworks that progressively weaken coordination bonds [5]. This effect becomes especially detrimental at high potential states where diminished electrostatic screening dramatically increases structural collapse susceptibility. Moreover, although the formation of irreversible compounds is suppressed in neutral electrolytes, the issue of manganese dissolution becomes more severe. Studies on Zn//MnO_2_ batteries with different polymorphs demonstrate that the charge storage mechanism involves substantial dissolution–deposition reactions [6] The dissolution of Mn-based cathode materials, particularly MnO_2_, is a critical factor contributing to capacity fade in aqueous Zn-ion batteries [12]. The chemical reductive dissolution of MnO_2_ during discharge is:

2 $${\mathrm{MnO}}_{2} + 4{\mathrm{H}}^{ + } + 2{\mathrm{e}}^{ - } \to {\mathrm{Mn}}^{2 + } + 2{\mathrm{H}}_{2} {\mathrm{O}}$$

These formed Mn^2+^ would be oxidized into ZnMn_2_O_4_ or ZnMn_3_O_7_ electrochemical inert species in presence of zinc hydroxides (Zn_4_SO_4_(OH)_8_) formed during discharging process. Researchers have attempted to suppress manganese dissolution by adding Mn^2+^ ions to the electrolyte, leveraging the shift in chemical equilibrium. Comparative studies on different Mn^2+^ concentrations have shown that although this strategy can temporarily inhibit Mn^2+^ dissolution and even contribute additional capacity through Mn^2^⁺ redeposition, a sharp capacity drop occurs after a certain number of cycles as the Mn^2^⁺ in the electrolyte becomes depleted or accelerate the formation of ZnMn_2_O_4_ instead [13, 14]. Nevertheless, the formation of inert species is not the full reason for stability decay. In most literatures, despite the modification strategies, MnO_2_ showed poor cycle stability at high loading mass (> 20 mg cm^−2^) while can stably operate for over thousands cycles at low mass loading. To date, Ah-level Zn-MnO_2_ batteries can only operate for less than 200 cycles in neutral electrolyte. The huge discrepancy between long cycle stability of thin electrode and poor cycle stability when the cathode scaled up cannot be simply explained by the formation of inactive species. The inactive species ratio should be similar in both cases as they share a common formation mechanism. Besides cathode degradation by electrochemical inactive product formation, we hypothesize that electroactive but insulating product accumulation may be another reason for fast capacity decay.

Acidic MnO_2_ cathode has been proposed and consider as one of the most attractive ways to fully utilize the two-electron transfer of Mn^4+^/Mn^2+^ [7] Attributing to the simple dissolution/deposition mechanism, this acidic MnO_2_//Zn battery is relatively stable than basic electrolyte and neutral electrolyte. Nevertheless, this kind of MnO_2_ cathode still suffers from incomplete dissolution, which produces dead MnO_2_ on current collector.

### Degradation of Vanadium-Based Cathodes

Vanadium-based material is another widely studied cathode for ZIBs due to their high specific capacity. They can be generally classified into two categories: oxides and phosphate. In aqueous electrolyte, the most serious problem is the high valent vanadium cation chemically/electrochemically reacts with water, resulting in dissolution and phase transformations during rest and charge–discharge cycling [8]. This would convert highly crystallized cathode materials into amorphous vanadium oxides or layered zinc vanadates, driven by oxidation to V^5+^ and interactions with protons or electrolyte components.

3 $$5{\mathrm{V}}_{2} {\mathrm{O}}_{5} + 3{\mathrm{H}}_{2} {\mathrm{O}} \to [{\mathrm{V}}_{10} {\mathrm{O}}_{26} ({\mathrm{OH}})_{2} ]^{4 - } + 4{\mathrm{H}}^{ + }$$

The transformation typically follows a dissolution–precipitation pathway, where vanadium species transformed to water-soluble intermediates like decavanadates ([V_10_O_26_(OH)_2_]^4−^) before reprecipitating as hydrated oxides, a process heavily influenced by pH fluctuations during cycling [2]. The cycled vanadium cathodes often exhibit low crystallinity, complicating characterization but sometimes improving ion diffusion and cyclability [9]. While the process enhances specific capacity by activation of solid-state oxide, it may also lead to voltage decay and structural instability by active material dissolution. This dynamic behavior results in the complex trade-offs between specific capacity and stability degradation in aqueous vanadium-based cathodes.

Compared with vanadium oxides, vanadium phosphates are relatively stable by stronger PO_4_^3−^–V^5+^ bond. Nevertheless, besides dissolution mechanism, the most significant characteristics of phosphate degradation is voltage decay. Although this degradation usually results in capacity increase while, the overall energy density is seriously reduced due to lower operation voltage. The voltage fading of vanadium phosphate is primary due to the structural intermixing of PO_4_^3−^ of the layered structure, which could be regulated by higher operation potential or intercalation of organic molecules [10–12].

### Degradation of Polyhalides

**Fig. 3:**
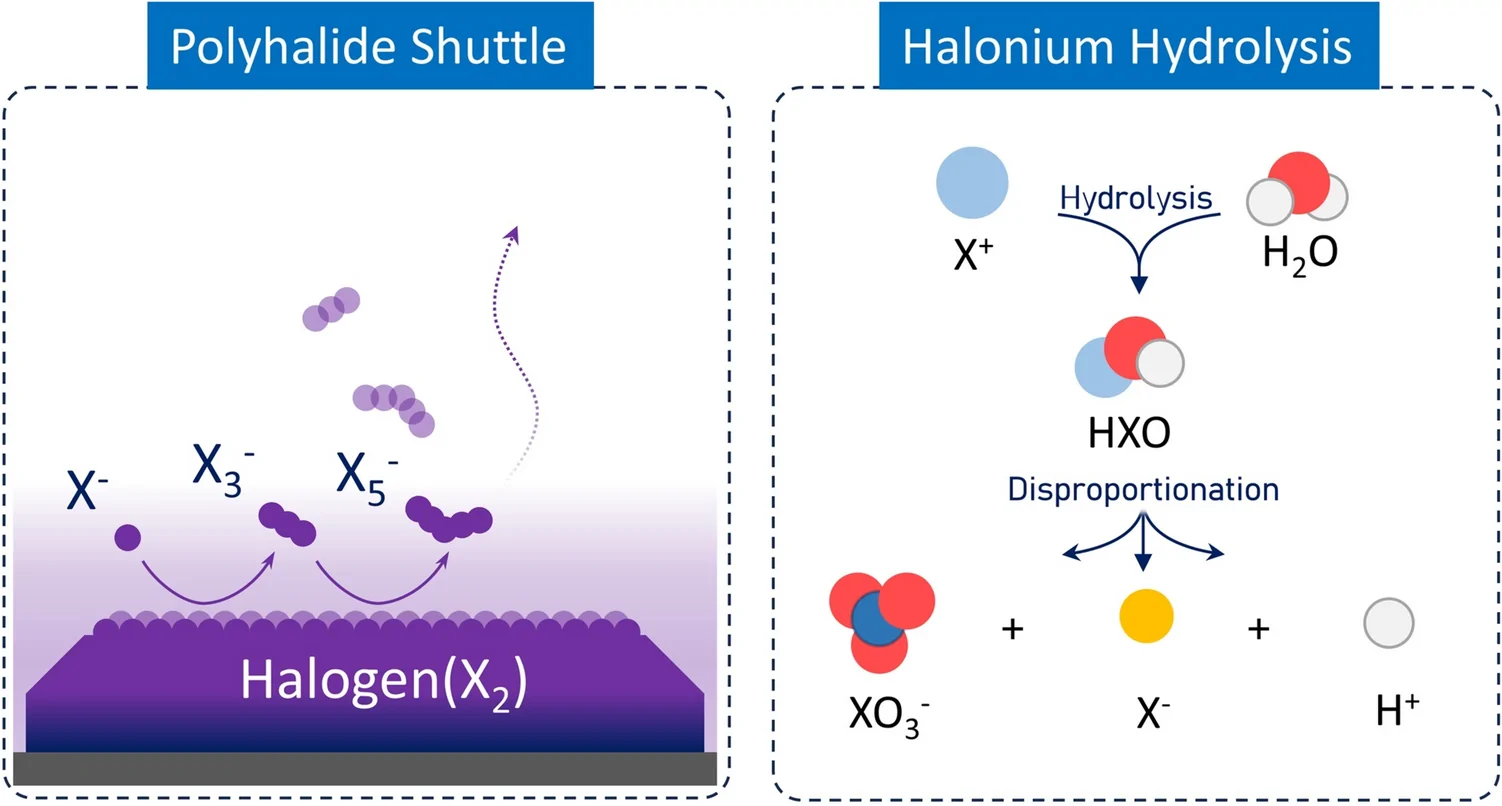
Degradation of halogen cathodes: polyhalides shuttle effect and hydrolysis of halonium

Halogen-based cathode materials (e.g., I_2_, Br_2_, Cl_2_) with large capacity, high voltage, as well as low cost are promising cathode materials for zinc batteries (Fig. 3). However, halogen-based cathodes suffer from critical challenges of shuttling effects and hydrolysis [13]. During discharge, halogens (X_2_) undergo reduction to form soluble polyhalide intermediates (X_3_^−^, X_5_^−^):

4 $${\mathrm{X}}_{2} + 2{\mathrm{e}}^{ - } \rightleftharpoons 2{\mathrm{X}}^{ - } \left( {{\mathrm{Solid}} - {\mathrm{phase}}\,{\mathrm{reduction}}} \right){ }$$

5 $${\mathrm{X}}_{2} + {\mathrm{X}}^{ - } \rightleftharpoons {\mathrm{X}}_{3}^{ - } \left( {{\mathrm{Dissolution}}\,{\mathrm{equilibrium}}} \right)$$

The chemical bonding between Lewis basic halide ion and halogen element with Lewis acidity is a spontaneous reaction. The large polyhalide ions are typical soft bases which has low affinity toward metal cations. Therefore, the polyhalide salts can be easily ionized and dissolved in the aqueous electrolyte, which lead to shuttle effect. The highly oxidative polyhalides would subsequently corrode the zinc anode by parasitic reactions (X_3_^−^ + Zn → Zn^2+^  + 3X^−^), which results in low cycle stability as well as serious self-discharge. Besides shuttle effect, halogens also suffer from hydrolysis. Hydrolysis reactions (e.g., I_2_ + H_2_O → HOI + H^+^  + I^−^) further reduce the cycle life of halogen cathodes, especially for light halogen such as Cl_2_- and Br_2_-based cathode in neutral/alkaline electrolytes [14]. For two-electron process (I^−^/I^0^/I^+^) in high-voltage zinc–halogen batteries, the active halonium species is particularly prone to hydrolyze, for which the process is electrochemical irreversible. While strategies like halogen confinement in porous hosts or electrolyte additives can mitigate these issues, achieving long-term stability without sacrificing capacity remains a key research frontier for aqueous halogen-based batteries.

### Degradation of Polysulfides

Aqueous sulfur batteries (ASBs) have emerged as a promising candidate for next-generation energy storage systems due to their high theoretical specific capacity (1675 mAh g^−1^), low cost, and environmental friendliness. However, the sulfur cathode tends to generate soluble polysulfides during charge–discharge processes, leading to the notorious shuttle effect [15]. This phenomenon results in active material loss, rapid capacity fading, and reduced Coulombic efficiency, significantly deteriorating battery performance.

$${\mathrm{S}}_{4}^{2 - } + 4{\mathrm{H}}_{2} {\mathrm{O}} + 6{\mathrm{e}}^{ - } \rightleftharpoons 4{\mathrm{HS}}^{ - } + 4{\mathrm{OH}}^{ - }$$

6 $${\mathrm{E}}^{0} = 0.51_{{}} {\mathrm{V}}\left( {{\mathrm{vs}}_{{}} {\mathrm{SHE}}} \right)$$

$${\mathrm{S}}_{4}^{2 - } + 4{\mathrm{H}}_{2} {\mathrm{O}} + 6{\mathrm{e}}^{ - } \rightleftharpoons 4{\mathrm{HS}}^{ - } + 4{\mathrm{OH}}^{ - }$$

7 $${\mathrm{E}}^{0} = 0.51_{{}} {\mathrm{V}}\left( {{\mathrm{vs}}_{{}} {\mathrm{SHE}}} \right)$$

$${\mathrm{S}} + {\mathrm{H}}_{2} {\mathrm{O}} + 2{\mathrm{e}}^{ - } \rightleftharpoons {\mathrm{HS}}^{ - } + {\mathrm{OH}}^{ - }$$

8 $${\mathrm{E}}^{0} = 0.51_{{}} {\mathrm{V}}\left( {{\mathrm{vs}}_{{}} {\mathrm{SHE}}} \right)$$

9 $$3{\mathrm{S}}_{4}^{{2 - }} + 2{\mathrm{e}}^{ - } \rightleftharpoons 4{\mathrm{S}}_{3}^{{2 - }} \,{\mathrm{E}}^{0} = - 0.48\,{\mathrm{V}}\left( {{\mathrm{vs}}\,{\mathrm{SHE}}} \right)$$

10 $${\mathrm{S}}_{3}^{{2 - }} + {\mathrm{S}} \rightleftharpoons {\mathrm{S}}_{4}^{{2 - }} \,{\mathrm{E}}^{0} = - 0.48\,{\mathrm{V}}\left( {{\mathrm{vs}}\,{\mathrm{SHE}}} \right)$$

The solubility of polysulfides (PS) in aqueous solvents differs significantly from organic solvents. Short-chain PS (e.g., Li_2_S_n_, Na_2_S_n_, K_2_S_n_, n ≤ 4) exhibit high solubility in water, whereas long-chain PS dissolve poorly, leading to a simplified one-step solid-to-liquid (S−L) redox reaction in sulfur–alkali batteries (SABs) [16]. The high ionic conductivity (> 100 mS cm^−1^) in aqueous electrolytes accelerates mass transport. Licht et al. initially categorized this process into full (S–L) and half (L–L) routes, with recent studies introducing an S–S redox pathway. The L–L redox offers fast kinetics but limited capacity (1254 mAh g^−1^), while the S–L redox enables higher capacity by utilizing solid sulfur, albeit with slower kinetics due to solid phase activation. Still, SABs outperform Li-SOBs in redox kinetics. The S–S redox potential (E^0^) depends on counterions, influencing practical cell configurations. To fully exploit SABs, further optimization of electrochemical metrics (kinetics, thermodynamics) and resolution of existing challenges are essential. Therefore, developing effective strategies to suppress cathode dissolution and mitigate shuttle effects remains a fundamental prerequisite for the advancement of aqueous battery systems. Besides polysulfide dissolution in aqueous Li, Na K batteries, low reaction kinetics of ZnS in zinc–sulfur batteries is a serious problem. The solid–solid conversion of ZnS/S results in huge overpotential (> 0.5 V), which makes the operation voltage of Zn–S battery lower than 0.8 V even under low current densities.

### Structural Degradation of Prussian Blue

Structural stability is one of the most discussed issues for Prussian blue analogues cathode materials. The primary distortion mechanism involves J-T effects of Mn^3+^/Fe^3+^ containing frameworks. The degenerate e_g_ orbital configuration induces octahedral distortions, altering lattice parameters that disrupt ionic diffusion. These effects are exacerbated by high charge density of Zn^2+^ (0.74 Å), creating strong electrostatic interactions with host frameworks that weaken coordination bonds. Phase transformations introduce additional mechanical stresses. Cubic–tetragonal transitions in Prussian blue analogues and P2-O2 changes in layered structures cause 2%–15% volume change, propagating microcracks and active material delamination. These dimensional changes also facilitate component dissolution through solvation reactions. The coupled effects of J-T distortions, phase transitions, and coordination disruption collectively degrade performance through multiple pathways: distorted ion channels impede transport, mechanical instability accelerates material degradation, and diminished structural reversibility limits cycle life [5, 17, 18]. In low-vacancy, high-potassium-content K_x_Mn[Fe(CN)_6_]_γ_, K^+^ ions shift cooperatively from high-symmetry positions, leading to pronounced slide distortions and a phase transition from cubic to monoclinic [19, 20]. The resulting phase transitions during cycling induce volume changes and internal stress, ultimately causing capacity fading. During cycling, transitions between Mn^3+^ and Mn^2+^ lead to repeated phase switching, structural fatigue, and degradation. J–T distortions also reduce the electron transfer rate and the Mn redox potential, impairing rate capability.

In aqueous electrolyte, Prussian blue cathode materials face more serious structural distortion issues, primarily A-site cation slide and Jahn–Teller distortions. In aqueous environments, the presence of water intensifies these issues, as water can penetrate the framework, interact with distorted structures, and trigger side reactions or Mn dissolution. Therefore, it is crucial to suppress the water activity and hence to reduce the direct reaction between water molecule and A-site metal centers, which would reduce side reactions and hence the better long-term electrochemical stability. Moreover, A-site cation sliding can exacerbate J–T distortions by locally altering the octahedral geometry, and the two mechanisms may act synergistically to amplify lattice parameter changes, further destabilizing the framework and accelerating structural degradation.

### Structural Degradation of Ni(OH)_2_

**Fig. 4:**
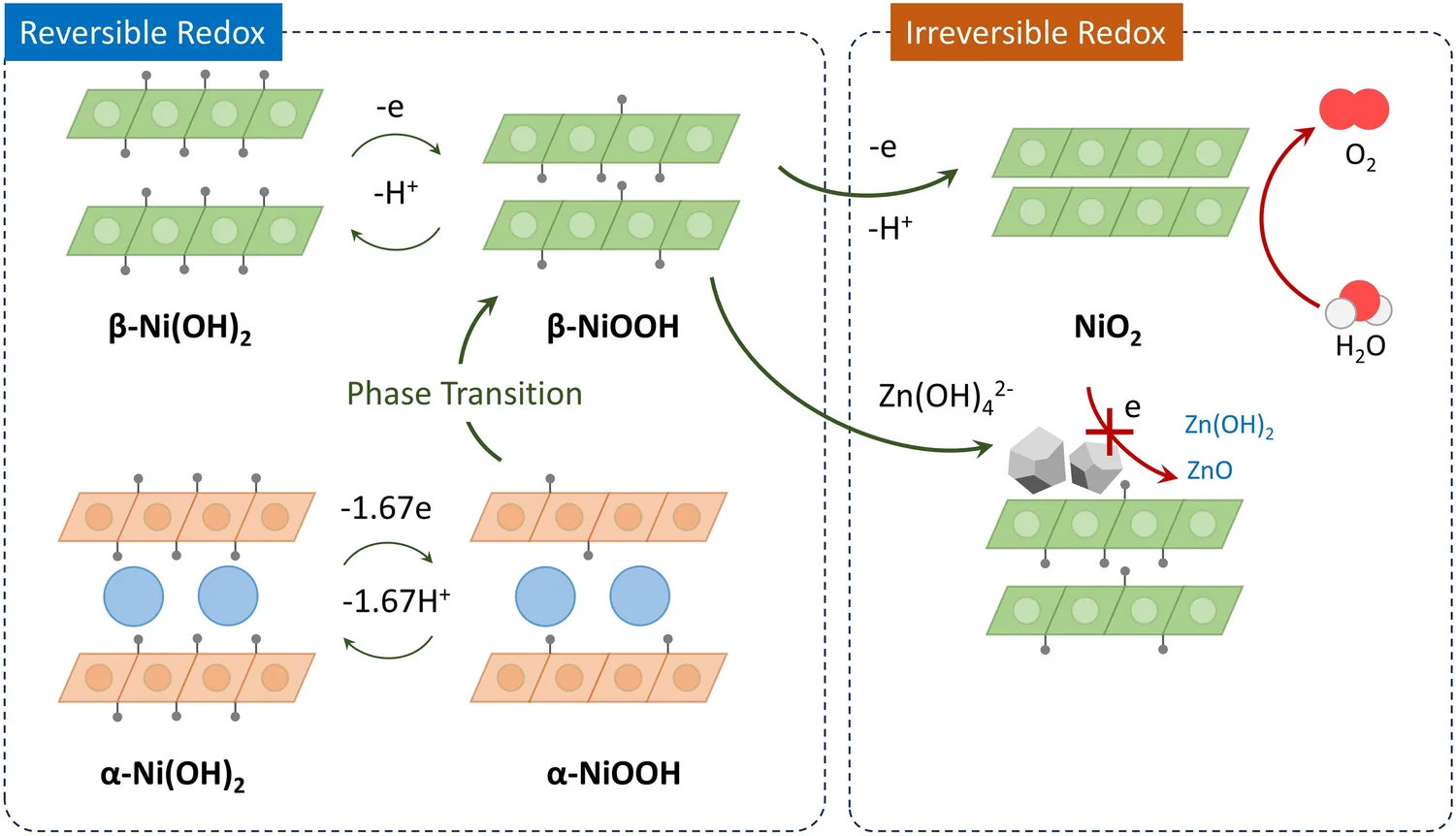
Redox mechanisms and degradation of Ni(OH)_2_ cathodes

Ni(OH)_2_-based cathodes are widely used in various kinds of secondary alkaline batteries such as Ni–Cd battery, Ni–Zn battery, and Ni-mMH battery. The electrochemical performance of nickel hydroxide (Ni(OH)_2_) as a cathode material in rechargeable batteries is significantly influenced by its structure. Perfectly crystalline Ni(OH)_2_ exhibits limited electrochemical activity, making it unsuitable for battery applications (Fig. 4). Instead, battery-grade Ni(OH)_2_ requires controlled structural distortions, such as stacking faults, intercalated species, and defects, to enhance proton diffusion and electronic conductivity. Ni(OH)₂ exists in two primary polymorphs: α-Ni(OH)_2_ and β-Ni(OH)_2_, each with distinct structural characteristics. β-Ni(OH)_2_ adopts a brucite-like layered structure with hexagonal close-packed OH^−^ ions and Ni^2+^ occupying octahedral sites. However, anisotropic bonding—strong iono-covalent interactions within layers and weaker electrostatic forces between them—-facilitates layer misalignment, leading to stacking faults that improve electrochemical activity. In contrast, α-Ni(OH)_2_ has a turbostratic structure with intercalated water molecules (Ni(OH)_2_·(0.5–0.7)H_2_O), expanding the interlayer spacing to ~ 8 Å. This hydrated phase offers higher theoretical capacity (456 mAh g^−1^ via the α ↔ γ transition) but suffers from instability in alkaline electrolytes, often dehydrating into β-Ni(OH)_2_. Structural disorder can be introduced through synthesis conditions. Chemical precipitation methods allow control over crystallinity by adjusting pH, temperature, and additives (e.g., Co, Al, or CO_3_^2−^). For instance, urea hydrolysis or low-temperature precipitation favors α-Ni(OH)_2_, while high-pH environments promote ordered β-Ni(OH)_2_. Electrochemical synthesis, on the other hand, enables precise tuning of the OH⁻/Ni^2+^ ratio, where values > 6 yield crystalline β-phase, whereas lower ratios (< 1) produce disordered α-phase. The impact of disorder on battery performance is multifaceted. While α-Ni(OH)_2_ delivers higher capacity, its volumetric energy density is compromised by low tap density (~ 0.75 vs. ~ 2.1 g mL^−1^ for β-phase). Moreover, γ-NiOOH (derived from α-Ni(OH)₂ oxidation) undergoes severe swelling, degrading electrode integrity [21, 22]. Conversely, β-NiOOH exhibits minimal volume changes, enhancing cycle life. Additives like Co or Y₂O₃ mitigate parasitic oxygen evolution and stabilize the γ-phase, further optimizing efficiency. Besides structural degradation of Ni(OH)_2_, oxygen evolution reaction (OER) is another reason for cathode instability. It is well known that nickel is an OER-active metal catalytic center, especially in alkaline environment. The oxygen generation not only reduces the cathodic faradaic efficiency, and the loss of water is detrimental to the battery cell, especially for lean-electrolyte condition. Moreover, the gas generated during battery operation would cause cell distortion and lead to loss of electric contact of battery components. Zinc species dissolved in the alkaline electrolyte would also cause cathode failure. The Zn(OH)_4_^2−^ ions undergoes dehydrogenation during discharging process. In summary, strategic manipulation of structural disorder in Ni(OH)_2_ is pivotal for advancing nickel-based batteries, offering a pathway to higher energy densities and longer lifespans in energy storage systems.

## Cathode Stabilization Strategies by Electrolyte Engineering

Aqueous batteries have high safety and environmental benefits unmatched by organic batteries, but their cathode materials also face many challenges during battery operation. In the previous section, we discussed failure mechanisms of cathode materials of aqueous batteries in detail including structural collapse by lattice expansion/contraction during repeatedly intercalation/deintercalation of cations in intercalation-type cathode materials; dissolution and shuttle effects of active species caused by formation of water-soluble oxidative products. Besides, the side reaction such as OER and hydrolysis of active materials also influenced on the stability of cathode materials of aqueous batteries.

Electrolyte engineering has long been one of the most effective solutions aiming at these problems. On the one hand, the high reactivity of water molecule is responsible for serious structural degradation; on the other hand, water acts as a good solvent to dissolve active material and facilitates shuttle effect. Therefore, electrolyte engineering could directly solve these problems.

### Bulk Electrolyte Modulation

#### Electrolyte Modulation for Oxide Cathodes

**Fig. 5:**
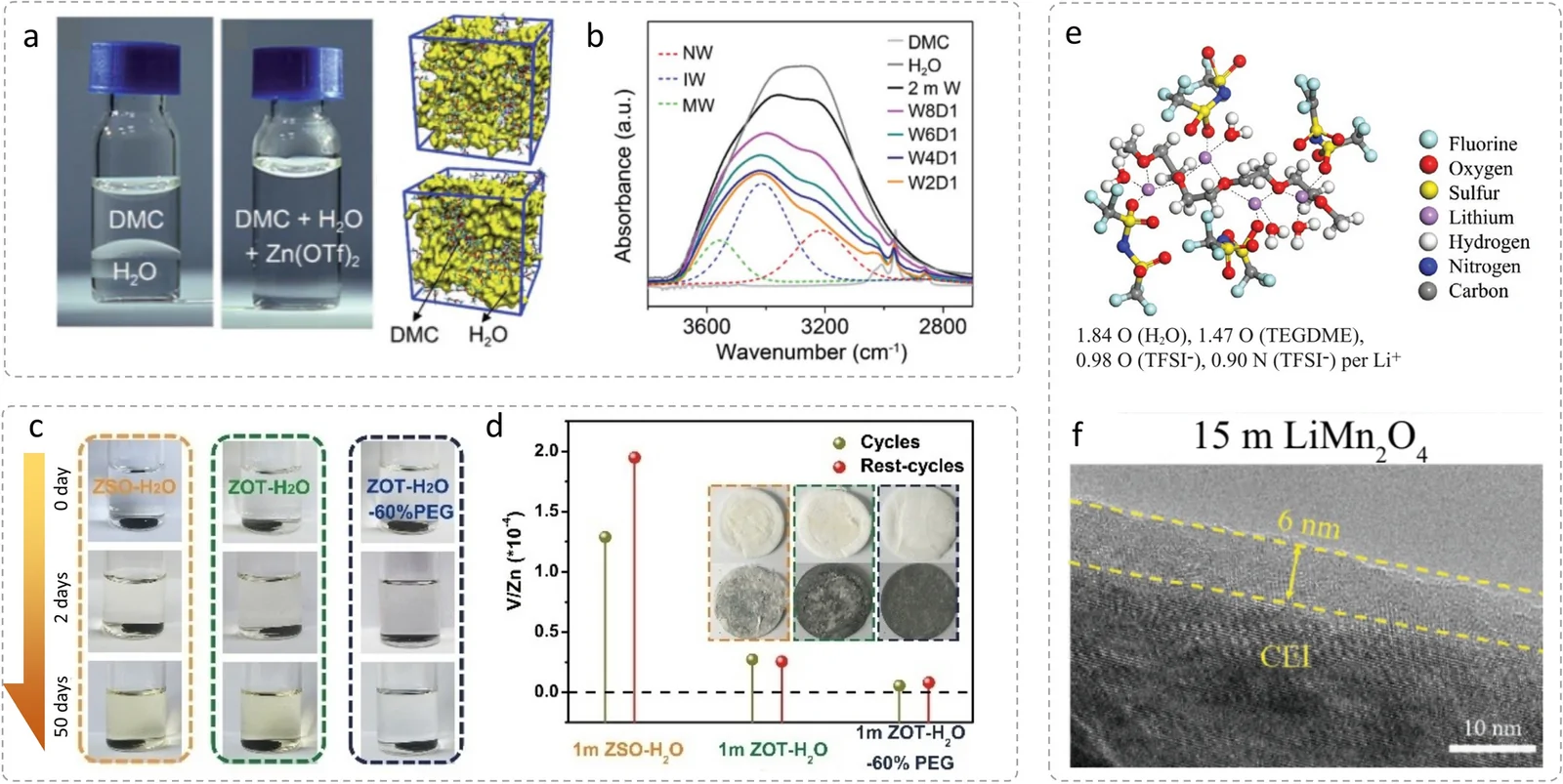
**a** Photographs and MD simulation 3D snapshot of phase separated DMC/H_2_O and homogenous phase when Zn(OTf)_2_ was added. **b** FTIR spectra of different DMC/H_2_O electrolytes [23]. **c** Dissolution of V_2_O_5_·nH_2_O cathode in ZnSO_4_, Zn(OTf)_2_ and Zn(OTf)_2_–60%PEG and **d** corresponding ICP-MS [24]. **e** Composition of the Li^+^ first coordination shell of EIWE and **f** CEI on cathode surface [25]

The main problems of aqueous batteries are the instability of the cathode material and the side reactions due to the high reactivity of water. Organic molecules can improve the interfacial stability of the cathode material by occupying the inner Helmholtz layer on the surface of the positive electrode material. In addition, organic molecules can regulate the solvation structure of the electrolyte through intermolecular interactions and inhibit the side reactions of water and negative electrode. Therefore, selecting a suitable organic co-solvent for the aqueous electrolyte can alleviate the dissolution problem of the cathode material, maintain the structural stability of the material and improve the performance of the electrolyte and the negative electrode. Water/organic hybrid electrolytes have been widely used in enhancing the stability of aqueous zinc-ion batteries. In summary, these studies demonstrate that introducing organic co-solvents to modulate the solvation structure is an effective strategy to suppress cathode dissolution (Fig. 5).

Dong et al*. *[23] added an organic solvent, dimethyl carbonate (DMC), to a low concentration aqueous solution of trifluoromethanesulfonic acid (Zn(OTf)_2_) and utilized the joint participation of DMC and OTf to adjust the solvated structure of Zn^2+^ to form a new solvated coating layer, which stabilizes the zinc negative electrode and is compatible with a variety of cathode materials, such as V_2_O_5_ and MnO_2_, and inhibits the dissolution of the cathode materials, thus improving the battery cycling performance. Meng et al*.* [26] prepared an acetonitrile–water co-solvent electrolyte by adding acetonitrile as an organic co-solvent to Zn(OTf)_2_ aqueous electrolyte. Acetonitrile can regulate the solvated structure of the electrolyte and inhibit the dissolution of vanadium, the active ingredient in vanadium-based anode materials (Al–V–O, V–O–H, etc.), thus improving the stability of the anode structure. In addition, acetonitrile can inhibit the decomposition and side reaction of water in the electrolyte, thus reducing the generation of by-products on the surface of the anode, keeping the ion transport channel unobstructed, and improving the cycling stability of the battery.

Chen et al*.* [24] introduced polyethylene glycol (PEG400) as a co-solvent into the aqueous electrolyte with low concentration of Zn(OTf)_2_. The hydroxyl group of PEG can form hydrogen bonds with water molecules, reduce the activity of free water molecules, and adjust the solvation structure of Zn^2+^, thus inhibiting the occurrence of side reactions such as dissolution of anodic vanadium and decomposition of water, and improving the cycling stability. Similarly, Chang et al. [27] also designed a hybrid aqueous–organic electrolyte with performance modulation by hydrogen bonding. By mixing ethylene glycol with aqueous zinc sulfate, the hydrogen bonding between ethylene glycol and water was used to regulate the solvation of water and Zn^2+^, which suppressed the side reactions on the surface of the cathode material, and the assembled batteries still had good cycling performance at low temperatures (− 20 °C). Mei et al. [28] also used organic co-solvents to control the solvation structure of Zn^2+^. They used triethyl phosphate (TEP) as an organic co-solvent to form an organic–water hybrid electrolyte with Zn(OTf)₂ and water, which on the one hand reduces the solvation degree of Zn^2+^ through the coordination of TEP with Zn^2+^ and inhibits the activity of water, thus reducing side reactions such as hydrogen evolution; at the same time, the dissolution of V_2_O_5_/MXene cathode material was significantly inhibited by adjusting the solvation structure, and the cathode material remained structurally intact after cycling.

Aqueous lithium-ion batteries are safer, relatively inexpensive, and more environmentally friendly than organic systems. However, the presence of water results in both a narrower electrochemical window and lower energy density, as well as making electrode materials susceptible to corrosion or dissolution (*e.g.*, vanadium-based cathode materials), resulting in shorter battery life. To combine the advantages of both, researchers have developed and investigated aqueous/organic hybrid electrolytes. Shang et al. [25] used tetraethylene glycol dimethyl ether (TEGDME) as a co-solvent dissolved in a high concentration of LiTFSI aqueous electrolyte to create a “water-in-ether” electrolyte for aqueous lithium-ion batteries. The ether oxygen atoms of TEGDME, water molecules in the aqueous solution, and the oxygen and nitrogen atoms of TFSI^−^ can coordinate with Li^+^, which can participate in the regulation of the solvation shell structure and stabilize the interfacial chemistry of the electrolyte, and the increase in the concentration of TFSI^−^ and TEGDME in the inner layer of the cathode–electrolyte—the inner layer of the Helmholtz layer—can displace the water molecules from the surface of the cathode material and inhibit the oxidation side reaction of the cathode, and the increase in the concentration of TFSI^−^ and TEGDME in the inner layer of the cathode–electrolyte–the inner layer of the Helmholtz layer—can inhibit the oxidation side reaction of the cathode and enhance the stability of the cathode and improve the stability of the cathode–electrolyte interface.

Aqueous potassium ion batteries are rich in resources, high in safety, and have better long-cycle performance. Although the current energy density still needs to be improved and the electrode materials and fluids have more limitations, it is expected to achieve a breakthrough in performance through the optimization of electrolyte, the development of electrode materials, and the regulation of interface. Among them, the optimization strategy of the electrolyte is similar to that of aqueous zinc-ion and lithium-ion batteries, which can protect the cathode material by lowering the molecular weight of water in the solvated shell on the surface of the cathode and at the same time inhibit the activity of water through some intermolecular interactions. For example, Xia et al*. *[29] introduced butanedinitrile (SCN) into potassium bisfluorosulfonimide (KFSI) as a hydrogen bonding moderator. On the one hand, the cyano group was used to form weak hydrogen bonds with water and weaken the strong hydrogen bonding network of free water, thus reducing side reactions such as water decomposition in the electrolyte and expanding the electrochemical stabilization window, as well as improving the high-voltage resistance of the electrolyte. On the other hand, SCN enters the solvated shell layer of K^+^, which can reduce the number of water molecules in the inner layer and inhibit the side reactions of the positive electrode due to the involvement of water. And SCN and FSI preferred to water for decomposition can form a dense interfacial layer on the surface of the positive electrode, which protects the positive electrode material and improves the battery stability.

Water activity is one of the most important reasons for cathode dissolution. The addition of specific electrolyte additives can regulate the solvation structure of Zn^2+^, by disrupting the hydrogen bond network between water molecules and reducing the activity of free water, thereby inhibiting the dissolution of cathode materials. For instance, triethyl phosphate (TEP) with a high donor number (DN value) is more likely to occupy the area around the internal solvation sheath of Zn^2+^ compared to water and at the same time form hydrogen bonds with water, reducing the reactivity of water with the V_2_O_5_ cathode and improving the stability of the system [30]. Mei et al. [28] further investigated the optimal ratio of this triethyl phosphate (TEP)/water mixed electrolyte and designed a V_2_O_5_/MXene composite cathode material to explore the zinc storage mechanism of V_2_O_5_/MXene in the electrolyte. Experimental data show that the Zn-V_2_O_5_ battery using the electrolyte with A Zn(OTf)_2_-TEP/H_2_O-80% ratio still has a capacity retention rate as high as 78.6% after 4000 cycles at a current density of 0.5 A g^−1^, which is superior to the performance of the vast majority of reported Zn-V_2_O_5_ batteries.

Similar to the mechanism of action of TEP, propylene carbonate (PC) forms a complex with Zn^2+^, reducing the number of active water molecules and the amount of H^+^ in the electrolyte, thereby lowering the destructive effect of H^+^ and H_2_O co-insertion on the cathode material during discharge–charge [31]. Meanwhile, the addition of PC also lowers the freezing point of the electrolyte and improves the low-temperature performance of the battery. Specifically, under the conditions of − 40 °C and 0.1 A g^−1^, the capacity retention rate after 300 cycles is as high as 100%. LiOTF also plays the same role [32]. By coordinating with Zn^2+^, it forms hydrogen bonds with water molecules, reduces the activity and quantity of water molecules, and prevents water molecules from forming hydrogen bonds with oxygen atoms in the cathode material, resulting in weakened V–O bond strength and even structural collapse.

It is not difficult to find that the core idea of the above examples is to avoid the dissolution of the cathode material by reducing the activity of water molecules. During the first charge and discharge of the battery, due to defects or inhomogeneity on the surface of the electrode material, the local electric field is enhanced, which in turn triggers the decomposition reaction of the electrolyte. The decomposition products generated by the reaction will deposit on the surface of the electrode material, forming a passivation layer, namely the solid electrolyte interface (SEI). Because the reduction potential of organic electrolytes is usually low and the ESW is narrow, the SEI film was initially thought to be generated only in organic electrolytes. However, with the continuous development of aqueous batteries, researchers have found that SEI can also form in aqueous electrolytes. Suo et al. [33] successfully formed the first aqueous SEI by changing the Li-solvation sheath structure through super-concentration, significantly expanding the ESW of the system from 1.23 to over 4.0 V, and successfully introducing the SEI into aqueous batteries. Because the phenomenon of performance deterioration caused by the dissolution of the cathode material in aqueous batteries is very common, this discovery has inspired researchers in aqueous batteries. How can a passivation layer be formed on the surface of the electrode material to protect the electrode material and stabilize the interface? The researchers suppressed cathode dissolution by constructing a cathode–electrolyte interface (CEI) on the cathode surface. Among them, adding electrolyte additives is the simplest and cheapest method. Many studies have proved that choosing the appropriate electrolyte additives can protect the cathode material from dissolving.

VC/H_2_O mixture significantly enhances the cycling stability and coulombic efficiency of Li_1.05_Cr_0.10_Mn_1.85_O_4_ in aqueous solutions by forming a protective film on the surface of electrode materials, preventing electrolyte decomposition and water penetration [34]. Furthermore, adding Mn(CF_3_SO_3_)_2_ to the Zn(CF_3_SO_3_)_2_ electrolyte enables the Zn-MnO_2_ battery to still provide a reversible capacity of 1550 mAh after 50 cycles, and the total energy density reaches 75.2 Wh kg^−1^ [35]. Adding Mn(CF_3_SO_3_)_2_ can replenish dissolved Mn^2+^. Additionally, the volume of the CF_3_SO_3_^—^anion is relatively large, which can reduce the solvation effect of water molecules on Zn^2+^ and facilitate the migration of Zn^2+^. In addition, a uniform and porous MnO_x_ nanosheet layer was formed on the surface of the cathode, preventing the structural damage of the cathode material and improving the cycling stability. The NMP molecule exhibits the highest HOMO energy level, so it shows the greatest tendency to adsorb on the positive electrode surface and induce the formation of CEI [36]. It was observed that the energy of LUMO showed a downward trend. Therefore, NMP molecules preferentially adsorbed on the surface of Zn and underwent solvation with Zn^2+^ at the interface. Electron transfer occurs between NMP molecules and the cathode material, further enhancing the adsorption effect and making the CEI layer more stable. The CEI layer prevents direct contact between the electrolyte and the cathode material, thereby inhibiting the dissolution of vanadium.

#### Electrolyte Modulation for Chalcogen Cathodes

Selenium has a theoretical capacity of 678 mAh g^−1^. The polarization voltage of Se cathode is relatively low compared with sulfur cathodes. Se cathode undergoes the same reversible conversion reaction whether in organic or aqueous electrolyte:

11 $${\text{Se }} + {\text{ Zn}}^{{{2} + }} + {\text{ 2e}}^{ - } \rightleftharpoons {\mathrm{ZnSe}}$$

Although Se cathode showed limited battery performance in aqueous electrolyte of 1 M ZnTFSI, the performance is significantly enhanced when PEG was used. The ZnTFSI/PEG/H_2_O electrolyte enabled the stable cycle of Zn-Se battery (1.5 V, 600 mAh g^−1^), which is comparable with that of in EMC organic electrolyte. Nevertheless, the platform contribution is lower for aqueous electrolyte, which needs further electrolyte modification [37].

Tellurium cathode has a theoretical capacity of 419 mAh g^−1^ in two-electron conversion manner (Te^0^/ZnTe), and 1260 mAh g^−1^ when tellurium is further oxidized to TeO_2_. However, Te^4+^ undergoes vigorous hydrolysis in water to form TeO_2_, resulting in irreversible loss of active substances and increased resistance to charge transfer. Zhi et al. [38] found that 30 m ZnCl_2_ electrolyte can achieve the Te^2−^/Te^0^/Te^4+^ six-electron reaction. The high-concentration ZnCl_2_ electrolyte reduced water activity and inhibited Te^4+^ hydrolysis. Besides, Cl^−^ as a charge carrier can drive the reversible conversion of Te^0^/Te^4+^. Therefore, a high-energy Zn-Te battery was achieved with specific capacity of 1223.9 mAh g^−1^and significantly reduced polarization voltage (0.39 V).

By designing suitable organic co-solvents, the solvated structure of the electrolyte can be regulated by intermolecular interactions, which can not only broaden the voltage window and inhibit side reactions such as water decomposition in the aqueous electrolyte but also optimize the interfacial chemistry and stabilize the anode structure. To some extent, it solves the bottleneck problem of traditional aqueous batteries. While maintaining the advantages of the high safety of aqueous batteries, it improves the energy density and cycle life of batteries, providing an important technology pathway for the next generation of cost-effective and safe energy storage systems.

### Electrolyte Additives

Besides mixed organic–aqueous electrolyte, electrolyte additives have also attracted much attention due to their advantages of high efficiency, simple process, and the ability to effectively improve the electrochemical performance of aqueous batteries. The small amount of usage would change the overall properties on various aspects. Although electrolyte additive strategy is most used to alleviate the zinc anode problems, the electrolyte additives also showed significant advantages in enhancing the cathode performance.

#### Additives for Halogen Cathodes

**Fig. 6:**
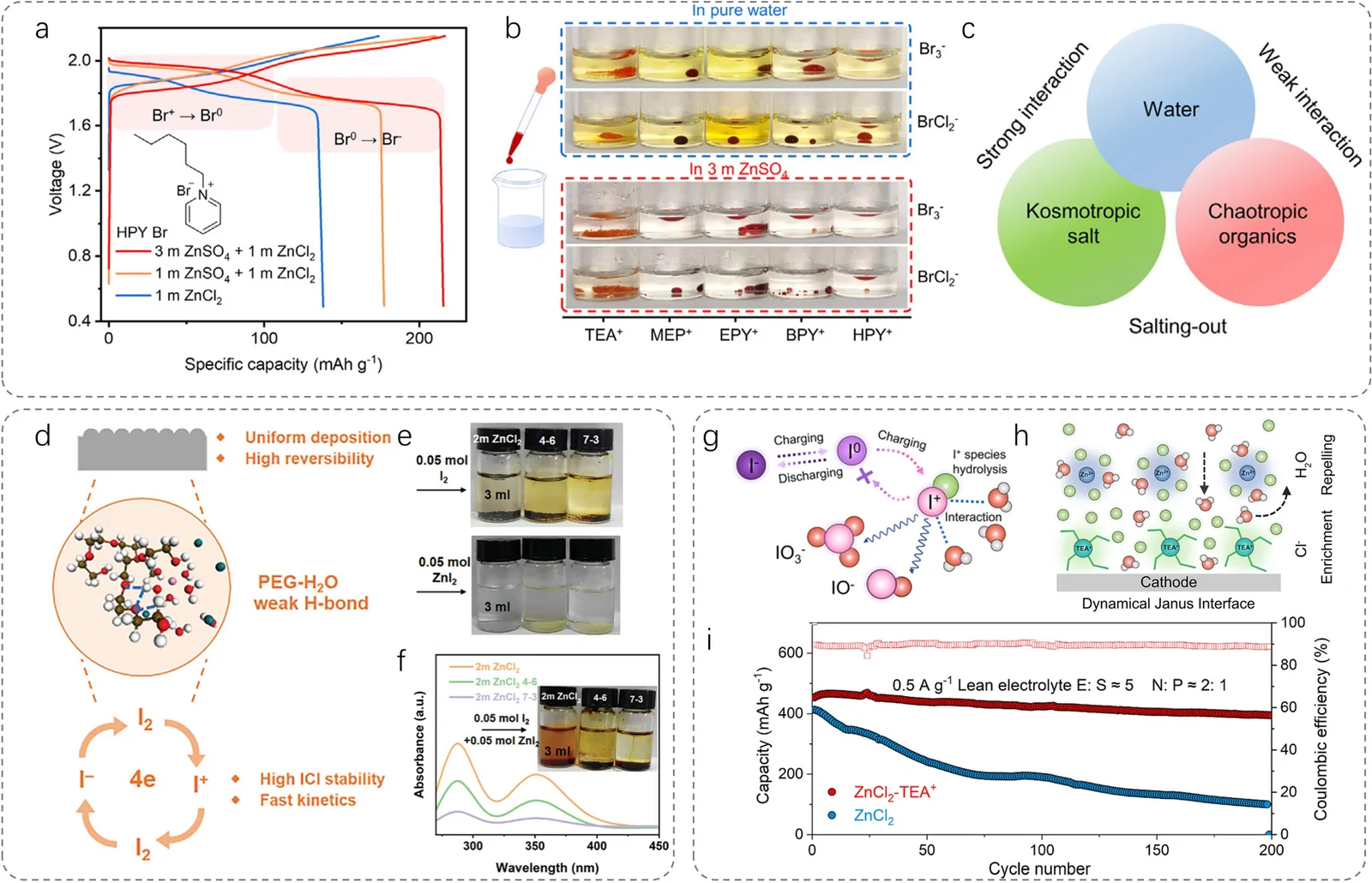
**a** Voltage profiles of the Zn-Br battery with HPY Br **b** Photographs of the complexes in pure water or ZnSO_4_ solution. **c** Salting-out effect of kosmotropic salt for chaotropic organics [41]. **d** PEG hybrid electrolytes and its influences on iodine redox. **e** Photographs of iodine species dissolution different electrolytes. **f** UV–Vis spectra of the generated I_3_^−^ anions in various electrolytes [42]. **g** Four-electron iodine conversion and hydrolysis of I^+^. **h** Janus interface in TEA^+^-containing systems **i** Cycling stability in ZnCl_2_ bare and ZnCl_2_-TEA^+^ electrolytes [43]

In aqueous zinc–halogen battery, the notorious shuttle effect will result in irreversible capacity loss as well as low cycle stability (Fig. 6). The traditional physical confinement strategies by porous matrix cannot fully solve this problem. Meanwhile, electrolyte additives could assist the inhibition of shuttle effects by the interaction between electrolyte additives molecule and polyhalide ions [39].

The mostly used electrolyte additives for halogen cathodes are quaternary ammonium salts (QASs). They stabilize polyhalide ions by the strong electrophilic nitrogen cations and hydrophobic alkyl chains. Through reasonable structural design, the shuttle effect of halogen cathodes can be effectively improved, and the stability of the system can be enhanced. Zhao et al. [40] introduced a series of QASs as electrolyte additives in dual-plating type zinc–iodine battery. The iodine forms conductive polyiodide ion liquid (PIL) cathode in presence of non-symmetric QASs, which enhanced the conductivity and suppressed polyiodides dissolution. The PIL cathode enables the zinc–iodine battery stably cycling for over 990 h at high areal capacity of 3.12 mAh cm^−2^. Beside iodine cathode, bromine cathode could also be stabilized by QASs. Liang et al*.* [41] used 1-hexylpyridinium bromide (HPYBr) as a complexing additive in electrolyte. Pyridine cations form stable complexes with polybromides, thereby inhibiting the shuttle effect of polybromides and stabilize bromonium cation in presence of HPYBr. The battery achieved a high specific capacity of 215 mAh g^−1^ with high capacity retention of 88.5% after 1000 cycles at a current density of 3C.

At present, high-concentration salt electrolytes and eutectic electrolytes were developed to inhibit the hydrolysis of I^+^ [42]. Although these strategies can reduce water activity, the charging process will still form water-rich zones, intensifying I^+^ hydrolysis and leading to irreversible conversion of four-electron iodine. The TEA⁺ dynamic Janus interface design proposed by He et al*.* [43] dynamically repels water and enriches Cl⁻ through the ion sieve effect, forming a unique water-poor and chlorine-rich interface. While inhibiting I⁺ hydrolysis, it maintains a high ionic conductivity, achieving an ultra-high rate of 189.5C**,** low-temperature operation at − 65 °C, and an ultra-long cycle of 35,000 times, breaking through the bottleneck of traditional strategies. Chen et al. [44] designed a bifunctional electrolyte additive trimethylamine hydrochloride (TAH), which contains both amine groups and Cl^−^. It can form a unique (TA)ICl bidentate coordination structure at low concentrations to achieve efficient anchoring of I^+^. DFT calculations show that this structure is more stable than the traditional single-tooth coordination (with a binding energy of − 3.20 eV), and it can be directly added to ZnSO_4_ aqueous solution, fully retaining the high ionic conductivity characteristics of aqueous electrolyte. The zinc–iodine battery showed a high capacity of 450 mAh g^−1^ at 2 A g^−1^, and an impressive rate performance of 200 mAh g^−1^ at 40 A g^−1^.

#### Additives for Chalcogen Cathodes

**Fig. 7:**
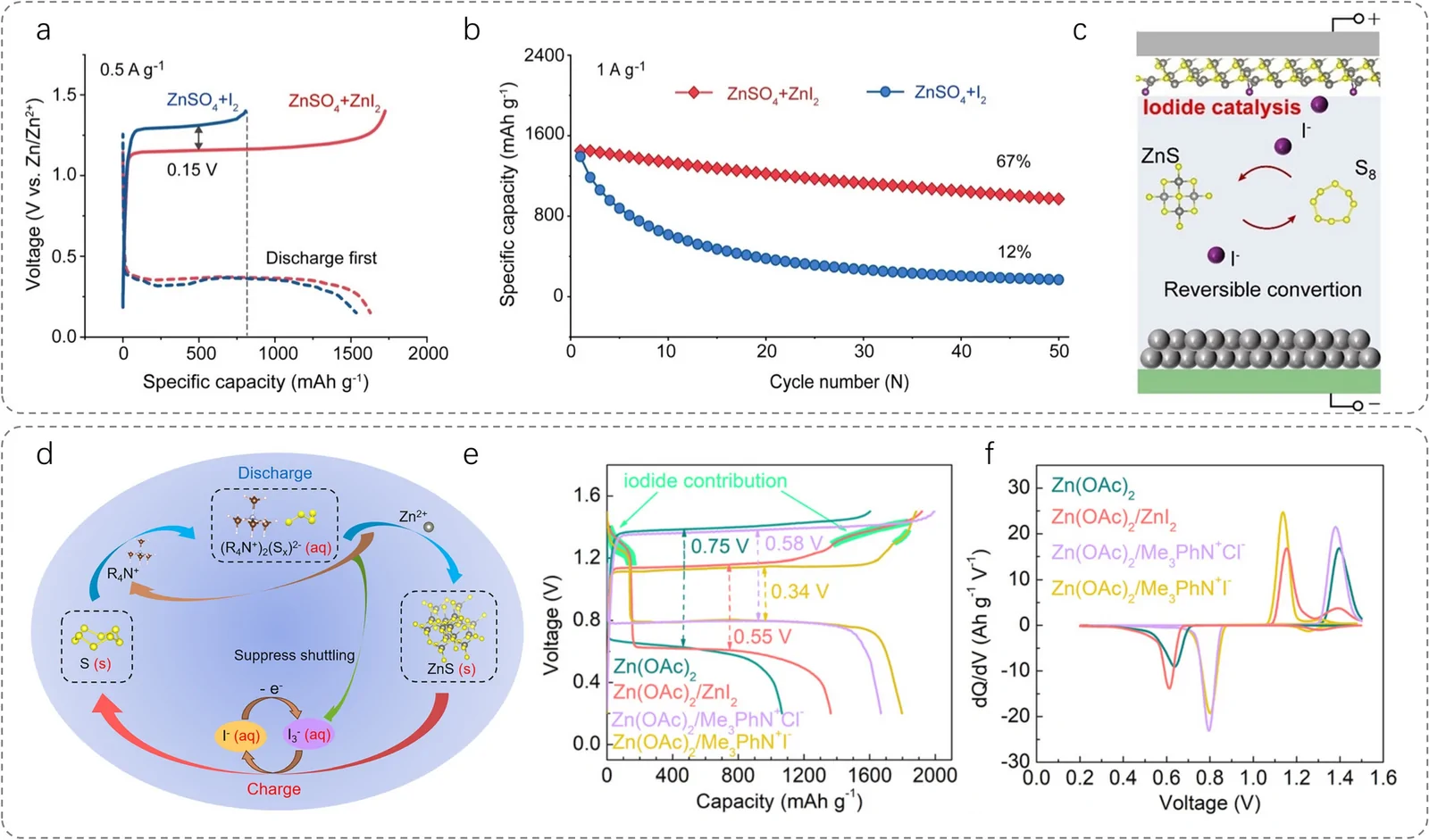
**a** First charge–discharge curves and **b** Cycling stability of the batteries **c** iodine catalytic mechanism [45]. **d** Sulfur reaction paths in aqueous Zn–S cells with the dual mediator of R_4_N^+^I^−^, **e** Galvanostatic charge–discharge and **f** dQ/dV curves with different electrolytes [46]

Recent studies have shown that high-capacity conversion-type cathode materials such as S (1148 mAh g^−1^) can be used in zinc batteries. Similar to halogen cathodes, sulfur cathodes generate soluble polysulfides during discharge, causing problems such as loss of active materials and low coulombic efficiency (Fig. 7). Moreover, the process of reducing sulfur (S_8_) to sulfide (S^2−^) involves multiple solid–liquid–solid phase transitions, with a high reaction energy barrier, resulting in low sulfur utilization rate and large voltage polarization.

In order to deal with the slow solid–solid conversion rate of ZnS and the poor redox kinetics of sulfur cathodes, researchers have found that adding iodine additives to the electrolyte can accelerate the solid–solid (S-ZnS) conversion reaction kinetics of sulfur cathodes. The strong interaction between I^−^ and ZnS rearranges the atomic structure of ZnS, weakening the Zn–S bond and promoting the electrochemical oxidation from ZnS to S. Enhance the capacity and stability of the battery [45]. Sun et al*.* [46] introduced a dual-mediating trimethylphenylammonium iodide (Me_3_PhN^+^I^−^) additive into the Zn(OAc)_2_ electrolyte to induce the formation of soluble polysulfide ion intermediates on cathode. This strategy transforms the solid–solid conversion reaction path between S and ZnS into a solid–liquid–solid conversion path. During the charging process, I_3_^−^/I redox pairs are utilized to promote the conversion reaction, and Me_3_PhN^+^ is employed to inhibit the shuttling of I_3_^−^. Experimental data show that the battery achieves a high discharge capacity of 1659 mAh g^−1^ at 0.1 A g^−1^ with low overpotential of 0.34 V. Under the conditions of a sulfur loading of 5.1 mg cm^−2^ and lean-electrolyte condition (10 μL mg_s_^−1^), the battery can still achieve a high discharge capacity of 1623 mAh g^−1^/8.3 mAh cm^−2^ with low overpotential of 0.37 V.

#### Additives for Oxide Cathodes

**Fig. 8:**
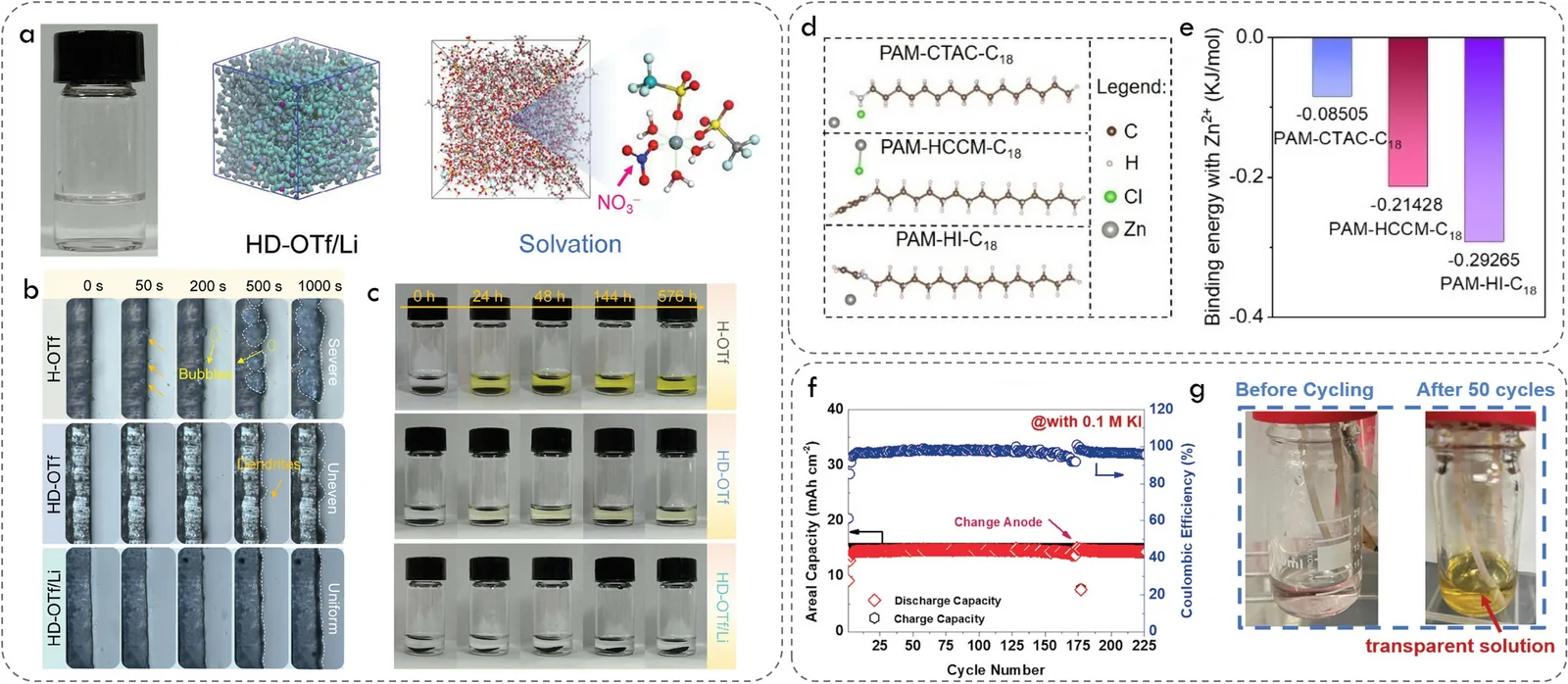
**a** Miscibility of the HD-OTf/Li electrolyte. **b** In situ electrochemical optical microscope images of the Zn surface and **c** Optical images of V_2_O_5_ electrodes immersed in H-OTf, HD-OTf, and HD-OTf/Li electrolytes [47]. DFT simulations of **d** the configuration and **e** the binding energy of PAM-CTAC-C_18_, PAM-HCCM-C_18_ and PAM-HI-C_18_ with Zn^2+^ [48]. **f–g** Cycling stability of the Zn-Mn^2+^/MnO_2_ cells with 0.1 M KI under 20 mA cm.^−2^ [49]

The long-term cycling stability of zinc–manganese battery systems in mild-acid aqueous electrolyte can also be modulated by electrolyte additives (Fig. 8). Guo et al*.* [50] found that the Na_0.44_MnO_2_ cathode with SDS additive had a higher initial discharge capacity, better rate performance, and cycle stability. Under the condition of 1 A g^−1^, the battery could still maintain 93% of its capacity after 1500 cycles. Zhang et al*.* [47] introduced dimethyl carbonate (DMC) and lithium nitrate (LiNO_3_) as additives into the aqueous solution of Zn(OTf)_2_ to construct the HD-OTf/Li composite electrolyte. Li^+^ and Zn^2+^ competed for coordination with water molecules, further reducing the content of free water molecules. It promotes the participation of NO_3_^−^ in the formation of the SEI film, effectively preventing the direct contact between active substances and electrolytes, and simultaneously effectively improving the mechanical problems of ion transport.

The quaternary ammonium salts and iodide ion additives mentioned above can not only improve the electrochemical performance of halogen cathodes but also effectively enhance the electrochemical performance of oxide cathodes. Zhi et al. [48] utilized the structural characteristics of cetylpyridine chloride (HCCM) to achieve ion selective regulation and cathode interface stabilization. The HCCM inhibits the dissolution and side reactions of MnO_2_ in the electrolyte and enhances the utilization rate of active substances. Through a unique three-step electrochemical energy storage process, it can achieve a high area capacity of 1.8 mAh cm^−2^ and a high specific energy of 0.858 Wh cm^−2^ even with a low MnO_2_ load mass of 0.5 mg cm^−2^.

For MnO_2_ in acidic electrolyte, Lu et al*.* [49] proposed a redox mediator strategy to enhance the cycling stability at high surface capacity by promoting the dissolution of MnO_2_ and restoring the “lost” capacity from the exfoliated MnO_2_. The iodide redox mediator continuously consuming the accumulated MnO_2_ by reducing dead MnO_2_. With low concentration iodide (0.1 M), the zinc–manganese static battery could stably cycle for more than 400 times at 2.5 mAh cm^−2^. The zinc–manganese flow battery stably cycled for more than 225 times at 15 mAh cm^−2^ and 50 times at 50 mAh cm^−2^.

#### Additives for PBA Cathodes

**Fig. 9:**
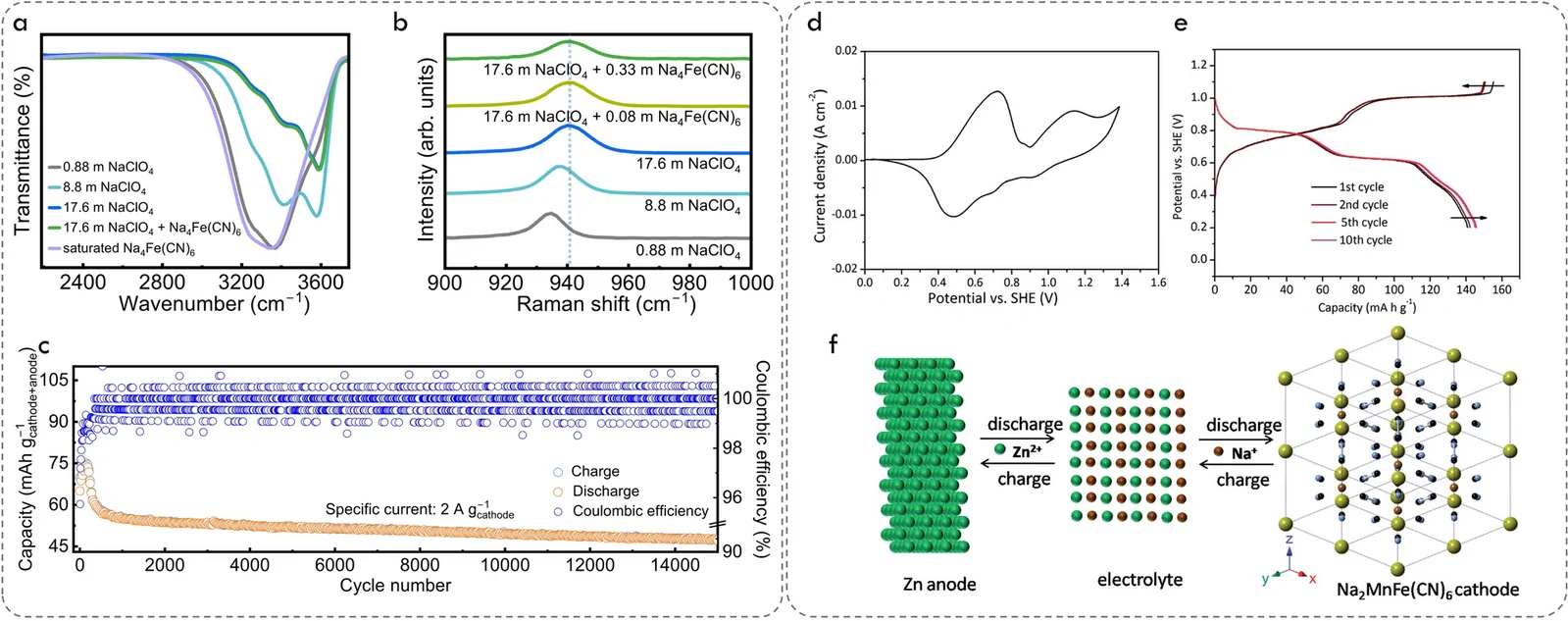
**a** FTIR spectra of the -OH stretching mode and **b** Raman spectra of ClO_4_^−^ stretching mode for different solutions. **c** Cycling performance of PTCDI||NaFeMnF cell in modified electrolyte [51]. **d** CV profiles of Na_2_MnFe(CN)_6_ cathode in electrolyte with added SDS. **e** Charge–discharge profiles in electrolyte with added SDS. **f** Schematic of the battery [52]

In the aqueous sodium-ion system, the traditional J–T distortion in Prussian blue cathode during charging and discharging causes irreversible structural damage, dissolution of Mn, and rapid capacity fading. In addition, the dissolved Mn^3^⁺ directly reacts with anode, generating by-products which will intensify polarization and accelerate capacity loss (Fig. 9). Wang et al*. *[51] added Na_4_Fe(CN)_6_ to a high-concentration electrolyte to capture the dissolved manganese during charging. The ferrocyanides fill the surface Mn vacancies formed in the Prussian blue cathode material during the cycling process, which effectively resolves the issues of unstable cathode structure and enhances cycling stability. The specific energy of the resulting aqueous sodium-ion battery was 94 Wh kg^−1^ at 0.5 A g^−1^, and the discharge capacity retention rate after 15,000 cycles at 2 A g^−1^ is 73.4%. Besides this strategy, Hou et al*.* [52] found that surfactants additives can stabilize the cathode interface by modulating solvation structure. For example, when sodium dodecyl sulfate (SDS) was added to the aqueous electrolyte to CMC, the ESW could reach 2.5 V. Through contact angle analysis, it was found that the contact angle of the electrolyte with SDS added on the positive electrode of Na_2_MnFe(CN)_6_ changed from 60° to 120°. The electrolyte adsorbed on the electrode surface through electrostatic interaction can effectively suppress the contact between the electrode and water. Further XRD analysis of the cathode after 2000 cycles revealed no change before and after the cycle, indicating that the addition of SDS can effectively improve the stability of the cathode material.

The application of electrolyte additives in aqueous batteries has made remarkable progress. By combining the different functionalities of different additives or designing multifunctional additives by molecular engineering, the goal of “one stone multi-birds” can be achieved. Moreover, the functionalities of more sustainable and cost-effective natural products additives are yet to be exploited. In the future, the research of electrolyte additives in aqueous batteries is expected to be more extensive and in-depth on multi-scales.

### Water-in-Salt Electrolyte

Traditional aqueous batteries using dilute aqueous electrolyte suffers from various problems induced by high reactivity of water molecules. Although electrolyte additives can improve the performance of the electrolyte, these additives cannot fully suppress the side reactions. Water-in-salt Electrolyte (WiSE) is developed to eliminate the drawbacks brought by water activity. In this kind of electrolyte, water activity is mostly suppressed by using extremely high concentration of salt. Depending on the solvent's ability to dissolve salts, the concentration of ultra-concentrated electrolytes is typically 3–5 m in organic solvents and 4–10 m in aqueous solvents, depending on the type of salt used [53]. Taking LiTFSI as an example, if its concentration exceeds 5 m, it can be called WiSE. When there is sufficient solvent water in the dilute solution, cations can form primary and secondary solvation sheath structures, and anions are distributed in the free water of the diffusion layer. Water molecules are mainly bonded with cations. In the secondary solvation sheath, the loosely bound water exists in the form of “free water”. As the salt concentration increases, the number of available water molecules decreases, and the solvation sheath structure of cations begins to change. When the concentration increases to the WiSE level, the average number of water molecules within the primary solvated sheath of the cation is significantly lower than the average number of water molecules in the dilute solution. Moreover, the cations no longer form the secondary solvation sheath structure. When the salt concentration increases again, the limited water molecules are insufficient to neutralize the electrostatic field generated by the cations, causing the anions to enter the primary solvation sheath structure, and its structure undergoes a complete change. This will lead to an increase in the interaction force between ions and a decrease in the interaction force between ions and solvents, and WiSE will thereby exhibit special physicochemical properties [54, 55].

As the salt concentration in the solution increases, the performance of WiSE also undergoes significant changes, such as pH, ESW, freezing point (T_f_), density, viscosity, and ionic conductivity. WiSE has successfully extended the ESW, a critical concern for electrochemists, from the initial 1.23 V to approximately 2–3 V. This advancement enables stable operation at high voltages while significantly mitigating the dissolution of electrode materials [56].

**Fig. 10:**
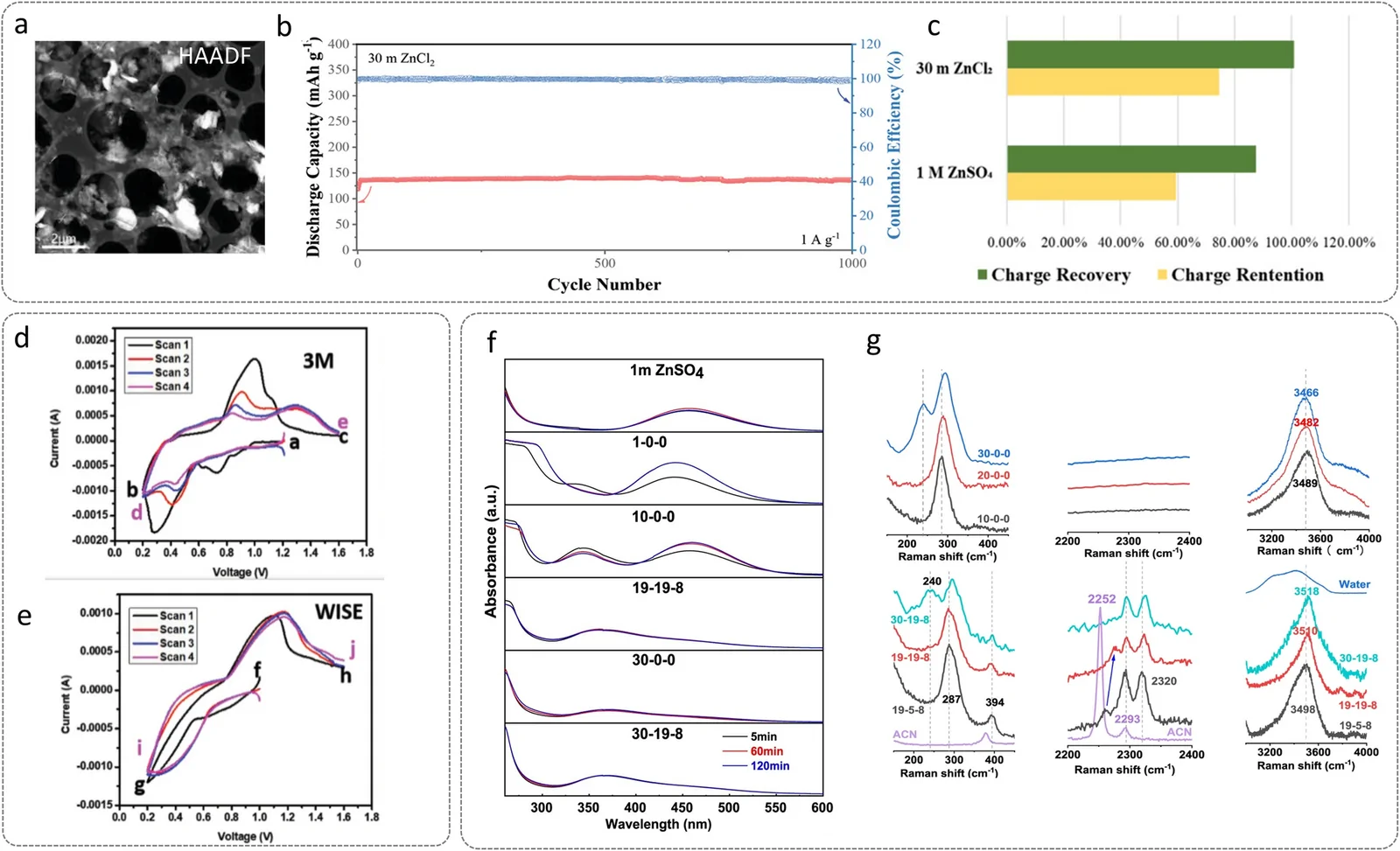
**a** HAADF image of the V_2_O_5_ electrode after discharge in WiSE. **b** Cycling stability of the V_2_O_5_ electrode at a current density of 1 A g^−1^ in WiSE. **c** Charge retention and charge recovery of the V_2_O_5_ electrode in different electrolyte environments at high temperatures [57]. **d**, **e** CV curves of 3 M ZnSO_4_ and 30 m ZnCl_2_ collected at different scan rates [58]. **f** Ultraviolet–visible spectra of ICl in different electrolyte systems vary with time. **g** Raman spectra of different electrolyte systems [59]

Since its proposal in 2015, WiSE has been successfully applied to various water-based battery systems, including lithium-ion batteries and zinc-ion batteries. Compared with traditional aqueous electrolytes, it has a wider electrochemical window, can support a working environment with higher voltages, and at the same time maintains the safety and low-cost advantages of aqueous electrolytes. Therefore, the problems of aqueous battery cathodes induced by water can be solved by WiSE (Fig. 10).

The single-salt system has become the most common system of WiSE due to its simplicity, ease of availability and low cost. However, since it contains only one metal salt, the performance of the electrolyte can only be optimized by adjusting the concentration of the salt. The optimization space is limited and it may not be possible to completely suppress the side reactions. Common salts include LiTFSI, ZnCl_2_, MgCl_2_, CaCl_2_, etc*.*

ZnCl_2_ is one of the most common and widely studied single-salt WiSE. It functions through multiple mechanisms in aqueous batteries, significantly enhancing battery performance, especially excelling in zinc-ion batteries (AZIBs). ZnCl_2_ was proposed as a widely used WiSE for zinc-ion batteries [60]. It was found that ZnCl_2_ WiSE inhibits dissolution of Ca_0.20_V_2_O_5_∙0.80H_2_O cathodes during cycling, which would formed highly soluble Zn_3_(OH)_2_V_2_O_7_·2H_2_O in dilute solution. Benefit from the high-concentration chloride ions that inhibits the dissolution of cathode materials. The cycle stability of the battery increases from 8.4% to 51.1% over 100 cycles even at a low current rate of 50 mA g^−1^ (C/10) [61]. V_2_O_5_ is a commonly used cathode material in aqueous zinc-ion batteries. Zn^2+^ and H_2_O co-intercalate into the V_2_O_5_ lattice in dilute electrolyte solutions, leading to lattice expansion, structural collapse, and loss of active substances, thereby deteriorating the electrochemical performance of the battery. Zhang et al*.* [61] discovered that ZnCl_2_ WiSE can effectively inhibit the co-intercalation of water molecules and solve the stability problem of vanadium oxide electrodes. When the electrolyte concentration was increased from 1 to 30 m, the specific capacity increased from 296 to 496 mAh g^−1^. The improvement of cycling stability (with a capacity retention rate of 90.5% after 300 cycles at 50 mA g^−1^) and the suppression of self-discharge (with a capacity retention rate of 74.66% after 300 h at 55 °C). Coincidentally, Tang et al. [57] also conducted research on this system, and the experimental data further confirmed that the WiSE strategy has a positive impact on battery performance, but the mechanism of action is different.

The concentration of ZnCl_2_ would significantly affect the redox potential of anion-accepting electrodes. Wu et al*. *[62] explored the electrochemical performance of Ferrocene in ZnCl_2_ electrolyte. On the one hand, the ZnCl_2_ WiSE minimized the dissolution of ferrocenium, on the other, increase the concentration of ZnCl_2_ would simultaneously increase the redox potential of cathode and decrease the redox potential in the anode, thereby increasing the overall battery voltage by 0.35 V. Inspired by this, Marschilok applied WiSE to the MoO_3_||Zn system [58]. Compared with the irreversible Zn_4_SO_4_(OH)_6_·3H_2_O formed by the traditional dilute solution, the reversible Zn_5_(OH)_8_Cl_2_·H_2_O protective layer effectively prevented Mo ions from migrating to the surface of the zinc anode, reducing the occurrence of side reactions. Meanwhile, due to the high insertion potential of H^+^ and Zn^2+^ in WiSE, the voltage of the battery rises, thereby increasing the energy density of the battery. After cycling 100 times at a current density of 100 mA g^−1^, the capacity retention rate of the WiSE battery is as high as 73%, which is three times higher than that of the dilute solution.

Although the single-salt WiSE system can effectively address issues such as electrode dissolution, narrow ESW, and side reactions in aqueous batteries, it also brings about problems such as excessive system viscosity and low ionic conductivity, resulting in poor performance of the battery in terms of rate performance, coulombic efficiency, and impedance. To address this issue, the performance of the electrolyte can be further optimized by adding other auxiliary salts or electrolytes for coordinated regulation.

Researchers have found that changing the dilute solution electrolyte of traditional zinc-iodine batteries to WiSE can reduce the activity of free water in the electrolyte, inhibit the dissolution of I_2_ and polyiodinated polymers, reduce the shuttling effect of iodine and improve the Coulombic efficiency (CE) [59]. Adding auxiliary salts to form a mixed electrolyte can not only provide a higher discharge capacity, but also improve the rate performance of the system. Ji et al*.* [63] added KI to the ZnCl_2_ WiSE, providing additional I^−^ ions to form stronger coordination bonds with Zn^2+^, thereby effectively inhibiting the dissolution of I_2_ and I_3_^−^ in the electrolyte and reducing the shuttle effect. Experimental data show that after 2000 cycles at 50 mA cm^−2^, excellent cycling stability with almost 100% capacity retention rate was achieved. Another strategy is to prepare a new type of WiSE with multiple solvation configurations. Zhao et al. [64] developed a novel WiDES electrolyte, which is composed of ZnCl_2_, choline chloride (ChCl), ethylene glycol (EG), and H_2_O, and exhibits multiple solvation configurations. In this system, ChCl not only serves as a Cl source but also acts as a hydrogen bond donor, forming a low eutectic solvent electrolyte with ZnCl_2_. This effectively lowers the melting point of the electrolyte and increases the solubility of ZnCl_2_, further reducing the activity of water. Furthermore, the interaction between the polar groups within EG and Zn^2+^ promoted the rearrangement of the solvated Zn^2+^ structure, and expanded the ESW from 3.65 to 4.02 V (vs. Ag/AgCl), and increased the coulombic efficiency of the first cycle from 68.2% to 98.7%. The experimental results show that at a low current density of 100 mA g^−1^, this electrolyte achieves a capacity of 987 mAh g^−1^, corresponding to an extremely high energy density of up to 1278 Wh kg^−1^, and the coulomb efficiency remains above 91.3%. This research achievement provides new ideas for the development of high-performance electrolytes and demonstrates its great potential in the field of energy storage.

In conclusion, WiSE is a highly promising aqueous battery electrolyte that significantly enhances the stability of the cathode in aqueous batteries and overcomes the limitations of traditional aqueous electrolytes. However, the development still needs to overcome some challenges. In the future, it is necessary to further optimize the salt concentration, additives, and interface design of WiSE to promote its application in more new battery systems.

### Hydrogel Electrolyte

**Fig. 11:**
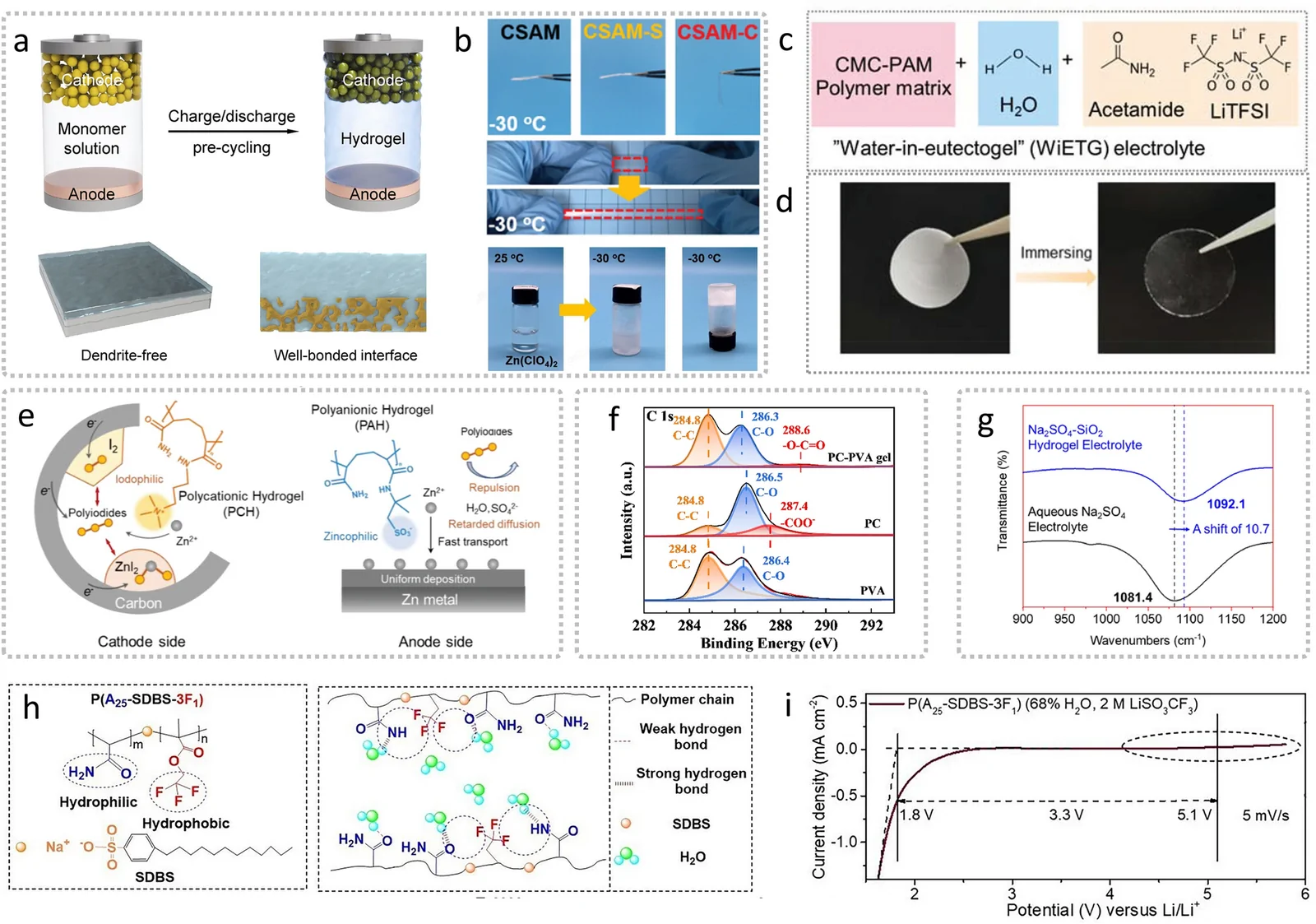
**a** Schematic of the cell fabrication by in situ electropolymerization with well-bonded interfaces [65]. **b** Digital images of the CSAM, CSAM-S, CSAM-C hydrogels, and Zn(ClO_4_)_2_ after 12 h storage at − 30 °C [66]. **c** Compositions of WiETG electrolytes. **d** Image of CMC-PAM polymer after freeze-drying and after immersing in ACE-131 electrolyte [67]. **e** Catalytic effect of PCH on iodine cathode and on Zn metal anode [68]. **f** XPS spectrum of C 1*s* of the PC-PVA, PC and PVA gel [69]. **g** FTIR of SO_4_^2−^ in aqueous Na_2_SO_4_ electrolyte and Na_2_SO_4_-SiO_2_ hydrogel electrolyte [72]. **h** Structure of HHE in P(A2_5_-SDBS-3F_1_) hydrogel. **i** Electrochemical stability window (ESW) of P(A_25_-SDBS-3F_1_) hydrogel electrolyte [70]

Hydrogel with structural rigidity of gel and the ionic conduction of liquid make it suitable for the electrolyte of aqueous batteries. Aiming at reducing structural degradation and active material dissolution, hydrogel-based electrolyte has unique ability to confine water molecule by polarity-tunable polymer chains. Therefore, both the water-induced cathode instability and shuttle effect would be efficiently suppressed. Moreover, by designing suitable functional groups, it is also possible to optimize the interfacial contact of cathode materials, reduce the interfacial impedance, and inhibit the formation of unstable by-products, thus improving the overall cycling stability of the battery (Fig. 11).

Chen et al*.* [71] prepared an all-round cellulose hydrogel using cotton as a raw material, tetraethoxysilane as a crosslinking agent, and glycerol as an antifreeze. For the cathode material MnO_2_, the adhesion of the hydrogel ensures that it is in close contact with the electrode surface and reduces the interfacial resistance, thus inhibiting the dissolution of the cathode material and maintaining the stable operation of the battery. Chen et al*.* [65] constructed a polyanion hydrogel by in situ electropolymerization method, which was also applied to Zn-MnO_2_ battery. On the anode side, the hydrogel formed in situ during the initial battery cycling can penetrate into the interior of the porous MnO_2_ anode and firmly bond with the interface of the anode material, thus reducing the charge transfer impedance and increasing the ion transfer efficiency, thereby accelerating the reaction kinetics. At the same time, the hydrogel can limit the activity of water in the electrolyte, thus inhibiting the dissolution of MnO_2_, maintaining the structural stability of the anode material, and thus inhibiting the capacity decay.

Hydrogen bonding enriched in hydrogels can also be designed to improve the performance of aqueous batteries. Huang et al*.* [66] designed a highly flexible polysaccharide hydrogel and modulated the performance of zinc-ion batteries based on the Hofmeister effect using a low concentration of Zn(ClO_4_)_2_ salts. The ClO_4_^−^, water, and polysaccharide polymer chains formed a ternary weak hydrogen bond. The ternary weak hydrogen bonding reduces the content of free water, which reduces the side reaction caused by the decomposition of water and the corrosion of the anode material polyaniline, and the surface of the anode can still be kept flat at − 30 °C, and the cycling stability has been greatly improved.

Modulation of functional groups of hydrogels is an effective method to suppress the polyiodide shuttle in zinc-iodide batteries. Yang et al*.* [68] designed a bilayer heterogeneous polyionic hydrogel linked by covalent bonding. The positive side of this hydrogel is a polycationic hydrogel with iodophilic groups, and the self-contained ammonium groups can immobilize the polyiodides by electrostatic action, effectively mitigating the shuttle effect. Meanwhile, the carbon nanotubes provide fast electron transfer to promote the redox kinetics of iodine species. On the negative side, a polyanion hydrogel exerts electrostatic repulsion on polyiodides and anions in the electrolyte, reduces corrosion and hydrogen precipitation reactions on the electrode surface, and inhibits the growth of zinc dendrites. The design realizes a long-life and high-kinetic aqueous zinc-iodide battery through the synergistic effect of heterogeneous polyanions. Xiong et al*.* [69] also prepared hydrogel electrolytes suitable for aqueous zinc-iodide batteries using polycarboxylate–polyvinyl alcohol and zinc trifluoromethanesulfonate. The abundance of polar oxygen-containing groups in the polycarboxylate–poly(vinyl alcohol) gels can trap polyiodides and mitigate the shuttle effect, thus improving the redox kinetics of I_2_/I^−^.

Besides common hydrogel composed of water as solvent, Hou et al*.* [67] prepared a new type of “water-containing eutectic gel,” combining carboxymethylcellulose-crosslinked polyacrylamide as a polymer matrix with deep eutectic solvent consisting of LiTFSI and acetamide. The designed hydrogel not only widens the electrochemical stabilization window and suppresses the oxidation side reactions of HER and TFSI, but also forms a uniform cathode–electrolyte interface on the cathode surface, suppresses the dissolution of cathode transition metals and OER, and improves the overall cycling stability. Li et al*. *[70] prepared a hydrophobic unit-conditioned hydrogel electrolyte by introducing a small number of hydrophobic particles into the hydrophilic polymer chain, supplemented with an amphoteric surfactant as a physical crosslinking agent. The hydrophobic units therein can enhance the hydrogen bonding strength between neighboring hydrophilic groups and water molecules, thus inhibiting water decomposition and also extending the electrochemical stabilization window.

Aqueous sodium-ion batteries are safe, inexpensive, and environmentally friendly, but they have poor low-temperature performance. The low-temperature performance of the batteries can be effectively improved by designing antifreeze hydrogel electrolytes. For example, Cheng et al*. *[72] used fumed silica as the gel matrix and methanol as the antifreeze additive to prepare a new type of Na_2_SO_4_-SiO_2_ hydrogel electrolyte, and the three-dimensional structure composed of SiO_2_, water and methanol solvent mixture can prevent the precipitation of Na_2_SO_4_ and the solidification of water, which can not only optimize the low-temperature performance of the battery, but also inhibit the oxygen precipitation reaction of the activated carbon cathode and improve the stability of the cathode material.

Non-metallic ammonium ions have the advantages of abundant reserves, low molar mass, and small radius of hydrated ions, which are easier to enhance the reaction kinetics and power density as charge carriers, and have recently been explored to be used as effective charge carriers in battery systems. Kuchena et al. [73] simply prepared a concentrated hydrogel electrolyte using xanthan gum as the polymer matrix, ammonium sulfate as the salt, and water as the solvent. The highly concentrated salt in this hydrogel reduces the dissolution of the cathode active substance NH_4_V_3_O_8_·2.9H_2_O and the side reaction caused by water, and the mechanical flexibility of the hydrogel can still maintain the contact between the cathode and the electrolyte when the battery is bent again to reduce the structural deformation. In addition, the high ionic conductivity of the hydrogel can accelerate the embedding/de-embedding of NH_4_^+^ in the layered vanadium oxides, thus improving the reaction kinetics of the anode. In addition, the adhesive property of xanthan gum can also improve the electrode/electrolyte interfacial contact and enhance the cycle life of the battery. Niu et al*.* [74–77] prepared a hydrogel with self-healing function using polyvinyl alcohol and ammonium chloride. The ionic conductivity and mechanical strength were balanced by a freeze/thaw strategy to form a crosslinked network structure and optimize the salt concentration of NH_4_Cl. The high ionic conductivity of the hydrogel accelerates the embedding/de-embedding of NH_4_^+^ and increases the capacity through the reversible Mn^4+^/Mn^3+^ redox reaction of the cathode active material MnO_2_. In addition, the hydrogel network reduces the dissolution of MnO_2_ and the flexibility of the hydrogel reduces the structural deformation of the anode, thus improving the cycling stability of the battery.

In general, for the cathode of aqueous batteries, hydrogel electrolytes can reduce the dissolution and side reactions of cathode materials by adjusting the activity and distribution of water molecules to enhance the stability; and their high hydrophilicity and strong hydrogen bonding network can also promote the embedding/de-embedding of protons or ions in the cathode, thus enhancing the capacity and cycling performance of the batteries. In addition, the quasi-solid nature of the hydrogel can be used to reduce the impedance at the anode-electrolyte interface and improve the kinetic performance. These properties make hydrogel electrolytes unique in improving the overall performance of aqueous batteries. Therefore, hydrogel electrolytes provide multiple performance advantages and represent an effective strategy to enhance both the cycling stability and energy density of aqueous batteries.

## Summary and Perspectives

We have summarized the failure mechanism of cathode materials induced by water molecules. For manganese-containing cathode, J-T distortion accelerated the Mn species dissolution, and the dissolved Mn is prone to redeposit as electrochemical inactive product or electronic insulating product in neutral/alkaline electrolyte. The incomplete reduction of MnO_2_ is the main reason for low Coulombic efficiency and low stability. For vanadium-based cathode, continuous dissolution of vanadium in aqueous solution causes the loss of active material. The structure change would also lead to voltage decay. Both of the fading mechanisms lead to a decay in energy efficiency. For Prussian blue analogues, the dissolution of active material induced by water attack is the primary reason for capacity fading. For chalcogen and halogen cathodes, insulating product and shuttle effects limit the capacity and detrimental to the cycle stability. For Ni(OH)_2_ cathodes, precipitation of zinc oxides at cathodes together with structure distortion is the primary capacity fading mechanism.

Electrolyte modulation could be one of the most efficient strategies to ameliorate the fading of the cathodes in different ways. Although various strategies have been proposed, several issues should be noted. Firstly, the amount of the electrolyte should be considered. In some of the strategies, the stabilization of cathodes is related with chemical reaction with the electrolyte itself. Under these circumstances, the ratio of the cathode materials and electrolyte greatly influenced the effectiveness of the modulation. Secondly, since electrolyte always in contact with both the cathode and anode materials, the influence of electrolytes on anode materials should also be considered. Lastly, it should be noted that when large amount of organic solvent was introduced into the electrolytes, the flammability of the electrolyte should be evaluated. There is a huge difference in flammability between water-in-organic and organic-in-water electrolytes.

In summary, electrolyte is one of the most important factors that influence the cathode performance. Electrolyte modulation has proven to be a highly effective strategy in addressing various cathode-related problems. Previous studies have proposed various strategies. Nevertheless, the commercialization of many types of aqueous batteries is still on the way since the poor cathode performance under practical conditions. Therefore, novel electrolyte modulation methods are expected to accelerate the development of aqueous batteries under commercialized standard. Future research could focus on low-cost natural product additives or develop synergistic strategies that combine bulk electrolyte modification with targeted additives, which may further enhance cathode stability and promote practical applications.

## References

[1] Y. Xu, G. Zhang, J. Liu, J. Zhang, X. Wang et al., Recent advances on challenges and strategies of manganese dioxide cathodes for aqueous zinc-ion batteries. Energy Environ. Mater. **6**(6), e12575 (2023). 10.1002/eem2.12575

[2] R. Sinha, X. Xie, Y. Yang, Y. Li, Y. Xue et al., Failure mechanisms and strategies for vanadium oxide-based cathode in aqueous zinc batteries. Adv. Energy Mater. **15**(14), 2404815 (2025). 10.1002/aenm.202404815

[3] L. Tang, H. Peng, J. Kang, H. Chen, M. Zhang et al., Zn-based batteries for sustainable energy storage: strategies and mechanisms. Chem. Soc. Rev. **53**(10), 4877–4925 (2024). 10.1039/D3CS00295K 38595056 10.1039/d3cs00295k

[4] G. Li, L. Sun, S. Zhang, C. Zhang, H. Jin et al., Developing cathode materials for aqueous zinc ion batteries: challenges and practical prospects. Adv. Funct. Mater. **34**(5), 2301291 (2024). 10.1002/adfm.202301291

[5] W.R. Robinson, High-temperature crystal chemistry of V_2_O_3_ and 1% chromium-doped V_2_O_3_. Acta Crystallogr. Sect. B Struct. Crystallogr. Cryst. Chem. **31**(4), 1153–1160 (1975). 10.1107/s0567740875004700

[6] Z. Li, Y. Li, X. Ren, Y. Zhao, Z. Ren et al., Elucidating the reaction mechanism of Mn^2+^ electrolyte additives in aqueous zinc batteries. Small **19**(38), 2301770 (2023). 10.1002/smll.202301770 10.1002/smll.202301770 37222115

[7] D. Chao, W. Zhou, C. Ye, Q. Zhang, Y. Chen et al., An electrolytic Zn-MnO_2_ battery for high-voltage and scalable energy storage. Angew. Chem. Int. Ed. **58**(23), 7823–7828 (2019). 10.1002/anie.201904174 10.1002/anie.201904174 30972886

[8] Z. Xing, G. Xu, J. Han, G. Chen, B. Lu et al., Facing the capacity fading of vanadium-based zinc-ion batteries. Trends Chem. **5**(5), 380–392 (2023). 10.1016/j.trechm.2023.02.008

[9] Y. Aniskevich, S.-T. Myung, Gains and losses in zinc-ion batteries by proton- and water-assisted reactions. Chem. Soc. Rev. **54**(9), 4531–4566 (2025). 10.1039/D4CS00810C 40162993 10.1039/d4cs00810c

[10] L. Hu, Z. Wu, C. Lu, F. Ye, Q. Liu et al., Principles of interlayer-spacing regulation of layered vanadium phosphates for superior zinc-ion batteries. Energy Environ. Sci. **14**(7), 4095–4106 (2021). 10.1039/D1EE01158H

[11] V. Verma, S. Kumar, W. Manalastas Jr., J. Zhao, R. Chua et al., Layered VOPO_4_ as a cathode material for rechargeable zinc-ion battery: effect of polypyrrole intercalation in the host and water concentration in the electrolyte. ACS Appl. Energy Mater. **2**(12), 8667–8674 (2019). 10.1021/acsaem.9b01632

[12] H.-Y. Shi, Y. Song, Z. Qin, C. Li, D. Guo et al., Inhibiting VOPO_4_⋅xH_2_O decomposition and dissolution in rechargeable aqueous zinc batteries to promote voltage and capacity stabilities. Angew. Chem. Int. Ed. **58**(45), 16057–16061 (2019). 10.1002/anie.201908853 10.1002/anie.201908853 31482627

[13] J. Hao, S. Zhang, H. Wu, L. Yuan, K. Davey et al., Advanced cathodes for aqueous Zn batteries beyond Zn^2+^ intercalation. Chem. Soc. Rev. **53**(9), 4312–4332 (2024). 10.1039/D3CS00771E 38596903 10.1039/d3cs00771e

[14] T. Zhang, Y. Zhao, Y. Feng, B. Wang, Y. Zhang et al., Aqueous-S vs organic-S battery: volmer-step involved sulfur reaction. J. Am. Chem. Soc. **147**(13), 11501–11510 (2025). 10.1021/jacs.5c01727 40114649 10.1021/jacs.5c01727

[15] J. Liu, W. Zhou, R. Zhao, Z. Yang, W. Li et al., Sulfur-based aqueous batteries: electrochemistry and strategies. J. Am. Chem. Soc. **143**(38), 15475–15489 (2021). 10.1021/jacs.1c06923 34510890 10.1021/jacs.1c06923

[16] J. Liu, C. Ye, H. Wu, M. Jaroniec, S.-Z. Qiao, 2D mesoporous zincophilic sieve for high-rate sulfur-based aqueous zinc batteries. J. Am. Chem. Soc. **145**(9), 5384–5392 (2023). 10.1021/jacs.2c13540 36809916 10.1021/jacs.2c13540

[17] X. Xu, F. Xiong, J. Meng, X. Wang, C. Niu et al., Vanadium-based nanomaterials: a promising family for emerging metal-ion batteries. Adv. Funct. Mater. **30**(10), 1904398 (2020). 10.1002/adfm.201904398

[18] L. Kou, Y. Wang, J. Song, T. Ai, W. Li et al., Mini review: strategies for enhancing stability of high-voltage cathode materials in aqueous zinc-ion batteries. Chin. Chem. Lett. **36**(1), 110368 (2025). 10.1016/j.cclet.2024.110368

[19] J. Cattermull, M. Pasta, A.L. Goodwin, Structural complexity in Prussian blue analogues. Mater. Horiz. **8**(12), 3178–3186 (2021). 10.1039/d1mh01124c 34713885 10.1039/d1mh01124c PMC9326455

[20] M. Fiore, S. Wheeler, K. Hurlbutt, I. Capone, J. Fawdon et al., Paving the way toward highly efficient, high-energy potassium-ion batteries with ionic liquid electrolytes. Chem. Mater. **32**(18), 7653–7661 (2020). 10.1021/acs.chemmater.0c01347

[21] Y. Ma, X. Song, W. Hu, J. Xiong, P. Chu et al., Recent progress and perspectives of advanced Ni-based cathodes for aqueous alkaline Zn batteries. Front. Chem. **12**, 1483867 (2024). 10.3389/fchem.2024.1483867 39659873 10.3389/fchem.2024.1483867 PMC11628261

[22] G. Fu, K. Chang, B. Li, E. Shangguan, H. Tang et al., High rate performance of surface metalized spherical nickel hydroxide *via in situ* chemical reduction. Electrochim. Acta **207**, 28–36 (2016). 10.1016/j.electacta.2016.04.165

[23] Y. Dong, L. Miao, G. Ma, S. Di, Y. Wang et al., Non-concentrated aqueous electrolytes with organic solvent additives for stable zinc batteries. Chem. Sci. **12**(16), 5843–5852 (2021). 10.1039/D0SC06734B 34168809 10.1039/d0sc06734b PMC8179661

[24] Y. Chen, S. Guo, L. Qin, Q. Wan, Y. Pan et al., Low current-density stable zinc-metal batteries *via* aqueous/organic hybrid electrolyte. Batter. Supercaps **5**(5), e202200001 (2022). 10.1002/batt.202200001

[25] Y. Shang, N. Chen, Y. Li, S. Chen, J. Lai et al., An “ether-In-water” electrolyte boosts stable interfacial chemistry for aqueous lithium-ion batteries. Adv. Mater. **32**(40), e2004017 (2020). 10.1002/adma.202004017 32876955 10.1002/adma.202004017

[26] C. Meng, W. He, Z. Kong, Z. Liang, H. Zhao et al., Multifunctional water-organic hybrid electrolyte for rechargeable zinc ions batteries. Chem. Eng. J. **450**, 138265 (2022). 10.1016/j.cej.2022.138265

[27] N. Chang, T. Li, R. Li, S. Wang, Y. Yin et al., An aqueous hybrid electrolyte for low-temperature zinc-based energy storage devices. Energy Environ. Sci. **13**(10), 3527–3535 (2020). 10.1039/D0EE01538E

[28] Y. Mei, Y. Liu, W. Xu, M. Zhang, Y. Dong et al., Suppressing vanadium dissolution in 2D V_2_O_5_/MXene heterostructures *via* organic/aqueous hybrid electrolyte for stable zinc ion batteries. Chem. Eng. J. **452**, 139574 (2023). 10.1016/j.cej.2022.139574

[29] M. Xia, H. Fu, K. Lin, A.M. Rao, L. Cha et al., Hydrogen-bond regulation in organic/aqueous hybrid electrolyte for safe and high-voltage K-ion batteries. Energy Environ. Sci. **17**(3), 1255–1265 (2024). 10.1039/D3EE03729K

[30] S. Liu, J. Mao, W.K. Pang, J. Vongsvivut, X. Zeng et al., Tuning the electrolyte solvation structure to suppress cathode dissolution, water reactivity, and Zn dendrite growth in zinc-ion batteries. Adv. Funct. Mater. **31**(38), 2104281 (2021). 10.1002/adfm.202104281

[31] D.-S. Liu, Y. Zhang, S. Liu, L. Wei, S. You et al., Regulating the electrolyte solvation structure enables ultralong lifespan vanadium-based cathodes with excellent low-temperature performance. Adv. Funct. Mater. **32**(24), 2111714 (2022). 10.1002/adfm.202111714

[32] S. Liu, J. He, D.-S. Liu, M. Ye, Y. Zhang et al., Suppressing vanadium dissolution by modulating aqueous electrolyte structure for ultralong lifespan zinc ion batteries at low current density. Energy Storage Mater. **49**, 93–101 (2022). 10.1016/j.ensm.2022.03.038

[33] L. Suo, D. Oh, Y. Lin, Z. Zhuo, O. Borodin et al., How solid-electrolyte interphase forms in aqueous electrolytes. J. Am. Chem. Soc. **139**(51), 18670–18680 (2017). 10.1021/jacs.7b10688 29186955 10.1021/jacs.7b10688

[34] I.B. Stojković, N.D. Cvjetićanin, S.V. Mentus, The improvement of the Li-ion insertion behaviour of Li_1.05_Cr_0.10_Mn_1.85_O_4_ in an aqueous medium upon addition of vinylene carbonate. Electrochem. Commun. **12**(3), 371–373 (2010). 10.1016/j.elecom.2009.12.037

[35] N. Zhang, F. Cheng, J. Liu, L. Wang, X. Long et al., Rechargeable aqueous zinc-manganese dioxide batteries with high energy and power densities. Nat. Commun. **8**, 405 (2017). 10.1038/s41467-017-00467-x 28864823 10.1038/s41467-017-00467-x PMC5581336

[36] K. Wang, F. Liu, Q. Li, J. Zhu, T. Qiu et al., An electrolyte additive for interface regulations of both anode and cathode for aqueous zinc-vanadium oxide batteries. Chem. Eng. J. **452**, 139577 (2023). 10.1016/j.cej.2022.139577

[37] Z. Chen, F. Mo, T. Wang, Q. Yang, Z. Huang et al., Zinc/selenium conversion battery: a system highly compatible with both organic and aqueous electrolytes. Energy Environ. Sci. **14**(4), 2441–2450 (2021). 10.1039/D0EE02999H

[38] J. Du, Y. Zhao, X. Chu, G. Wang, C. Neumann et al., A high-energy tellurium redox-amphoteric conversion cathode chemistry for aqueous zinc batteries. Adv. Mater. **36**(19), 2313621 (2024). 10.1002/adma.202313621 10.1002/adma.202313621 38316395

[39] Q. Yue, Y. Wan, X. Li, Q. Zhao, T. Gao et al., Restraining the shuttle effect of polyiodides and modulating the deposition of zinc ions to enhance the cycle lifespan of aqueous Zn–I_2_ batteries. Chem. Sci. **15**(15), 5711–5722 (2024). 10.1039/D4SC00792A 38638220 10.1039/d4sc00792a PMC11023047

[40] H. Zhao, D. Yin, Y. Qin, X. Cui, J. Feng et al., Highly electrically conductive polyiodide ionic liquid cathode for high-capacity dual-plating zinc–iodine batteries. J. Am. Chem. Soc. **146**(10), 6744–6752 (2024). 10.1021/jacs.3c12695 38422617 10.1021/jacs.3c12695

[41] C. Xu, C. Lei, P. Jiang, W. Yang, W. Ma et al., Practical high-energy aqueous zinc-bromine static batteries enabled by synergistic exclusion-complexation chemistry. Joule **8**(2), 461–481 (2024). 10.1016/j.joule.2023.12.023

[42] T. Liu, C. Lei, H. Wang, J. Li, P. Jiang et al., Aqueous electrolyte with weak hydrogen bonds for four-electron zinc–iodine battery operates in a wide temperature range. Adv. Mater. **36**(32), 2405473 (2024). 10.1002/adma.202405473 10.1002/adma.202405473 38837833

[43] W. Zong, J. Li, C. Zhang, Y. Dai, Y. Ouyang et al., Dynamical Janus interface design for reversible and fast-charging zinc–iodine battery under extreme operating conditions. J. Am. Chem. Soc. **146**(31), 21377–21388 (2024). 10.1021/jacs.4c03615 39046802 10.1021/jacs.4c03615

[44] M. Wang, Y. Meng, M. Sajid, Z. Xie, P. Tong et al., Bidentate coordination structure facilitates high-voltage and high-utilization aqueous Zn-I_2_ batteries. Angew. Chem. Int. Ed. **63**(39), e202404784 (2024). 10.1002/anie.202404784 10.1002/anie.202404784 38868978

[45] P. Hei, Y. Sai, C. Liu, W. Li, J. Wang et al., Facilitating the electrochemical oxidation of ZnS through iodide catalysis for aqueous zinc-sulfur batteries. Angew. Chem. Int. Ed. **63**(9), e202316082 (2024). 10.1002/anie.202316082 10.1002/anie.202316082 38196064

[46] W. Wu, S. Wang, L. Lin, H.-Y. Shi, X. Sun, A dual-mediator for a sulfur cathode approaching theoretical capacity with low overpotential in aqueous Zn–S batteries. Energy Environ. Sci. **16**(10), 4326–4333 (2023). 10.1039/d3ee01749d

[47] X. Zhang, Z. Deng, C. Xu, Y. Deng, Y. Jia et al., Electrolyte engineering *via* competitive solvation structures for developing longevous zinc ion batteries. Adv. Energy Mater. **13**(48), 2302749 (2023). 10.1002/aenm.202302749

[48] C. Li, H. Yuan, T. Liu, R. Zhang, J. Zhu et al., Distinguish MnO_2_/Mn^2+^ conversion/Zn^2+^ intercalation/H^+^ conversion chemistries at different potentials in aqueous Zn||MnO_2_ batteries. Angew. Chem. Int. Ed. **63**(22), e202403504 (2024). 10.1002/anie.202403504 10.1002/anie.202403504 38563637

[49] J. Lei, Y. Yao, Z. Wang, Y.-C. Lu, Towards high-areal-capacity aqueous zinc–manganese batteries: promoting MnO_2_ dissolution by redox mediators. Energy Environ. Sci. **14**(8), 4418–4426 (2021). 10.1039/d1ee01120k

[50] H. Guo, Z. Shao, Y. Zhang, X. Cui, L. Mao et al., Electrolyte additives inhibit the surface reaction of aqueous sodium/zinc battery. J. Colloid Interface Sci. **608**, 1481–1488 (2022). 10.1016/j.jcis.2021.10.085 34742067 10.1016/j.jcis.2021.10.085

[51] Z. Liang, F. Tian, G. Yang, C. Wang, Enabling long-cycling aqueous sodium-ion batteries *via* Mn dissolution inhibition using sodium ferrocyanide electrolyte additive. Nat. Commun. **14**, 3591 (2023). 10.1038/s41467-023-39385-6 37328496 10.1038/s41467-023-39385-6 PMC10275921

[52] Z. Hou, X. Zhang, X. Li, Y. Zhu, J. Liang et al., Surfactant widens the electrochemical window of an aqueous electrolyte for better rechargeable aqueous sodium/zinc battery. J. Mater. Chem. A **5**(2), 730–738 (2017). 10.1039/C6TA08736A

[53] Z. Khan, D. Kumar, X. Crispin, Does water-in-salt electrolyte subdue issues of Zn batteries? Adv. Mater. **35**(36), e2300369 (2023). 10.1002/adma.202300369 37220078 10.1002/adma.202300369

[54] L. Suo, O. Borodin, T. Gao, M. Olguin, J. Ho et al., “Water-in-salt” electrolyte enables high-voltage aqueous lithium-ion chemistries. Science **350**(6263), 938–943 (2015). 10.1126/science.aab1595 26586759 10.1126/science.aab1595

[55] C. Deriu, L. Fabris, A surface chemistry perspective on SERS: revisiting the basics to push the field forward. Chem. Soc. Rev. **54**(11), 5224–5247 (2025). 10.1039/D4CS01242A 40134302 10.1039/d4cs01242a PMC11937889

[56] Q. Zhang, Y. Ma, Y. Lu, L. Li, F. Wan et al., Modulating electrolyte structure for ultralow temperature aqueous zinc batteries. Nat. Commun. **11**, 4463 (2020). 10.1038/s41467-020-18284-0 32901045 10.1038/s41467-020-18284-0 PMC7479594

[57] X. Tang, P. Wang, M. Bai, Z. Wang, H. Wang et al., Unveiling the reversibility and stability origin of the aqueous V_2_O_5_–Zn batteries with a ZnCl_2_ “water-in-salt” electrolyte. Adv. Sci. **8**(23), 2102053 (2021). 10.1002/advs.202102053 10.1002/advs.202102053 PMC8655202 34665530

[58] L. Wang, S. Yan, C.D. Quilty, J. Kuang, M.R. Dunkin et al., Achieving stable molybdenum oxide cathodes for aqueous zinc-ion batteries in water-in-salt electrolyte. Adv. Mater. Interfaces **8**(9), 2002080 (2021). 10.1002/admi.202002080

[59] Y. Zou, T. Liu, Q. Du, Y. Li, H. Yi et al., A four-electron Zn-I_2_ aqueous battery enabled by reversible I^-^/I_2_/I^+^ conversion. Nat. Commun. **12**(1), 170 (2021). 10.1038/s41467-020-20331-9 33419999 10.1038/s41467-020-20331-9 PMC7794333

[60] C. Zhang, J. Holoubek, X. Wu, A. Daniyar, L. Zhu et al., A ZnCl_2_ water-in-salt electrolyte for a reversible Zn metal anode. Chem. Commun. **54**(100), 14097–14099 (2018). 10.1039/C8CC07730D 10.1039/c8cc07730d 30488907

[61] L. Zhang, I.A. Rodríguez-Pérez, H. Jiang, C. Zhang, D.P. Leonard et al., ZnCl_2_ “water-in-salt” electrolyte transforms the performance of vanadium oxide as a Zn battery cathode. Adv. Funct. Mater. **29**(30), 1902653 (2019). 10.1002/adfm.201902653

[62] X. Wu, Y. Xu, C. Zhang, D.P. Leonard, A. Markir et al., Reverse dual-ion battery *via* a ZnCl_2_ water-in-salt electrolyte. J. Am. Chem. Soc. **141**(15), 6338–6344 (2019). 10.1021/jacs.9b00617 30917652 10.1021/jacs.9b00617

[63] Y. Ji, J. Xie, Z. Shen, Y. Liu, Z. Wen et al., Advanced zinc–iodine batteries with ultrahigh capacity and superior rate performance based on reduced graphene oxide and water-in-salt electrolyte. Adv. Funct. Mater. **33**(10), 2210043 (2023). 10.1002/adfm.202210043

[64] J. Zhao, Y. Chen, M. Zhang, Z. An, B. Nian et al., Iodine/chlorine multi-electron conversion realizes high energy density zinc-iodine batteries. Adv. Sci. **12**(1), 2410988 (2025). 10.1002/advs.202410988 10.1002/advs.202410988 PMC11714215 39499723

[65] L. Chen, T. Xiao, J.-L. Yang, Y. Liu, J. Xian et al., *In-situ* spontaneous electropolymerization enables robust hydrogel electrolyte interfaces in aqueous batteries. Angew. Chem. Int. Ed. **63**(21), e202400230 (2024). 10.1002/anie.202400230 10.1002/anie.202400230 38520070

[66] S. Huang, L. Hou, T. Li, Y. Jiao, P. Wu, Antifreezing hydrogel electrolyte with ternary hydrogen bonding for high-performance zinc-ion batteries. Adv. Mater. **34**(14), e2110140 (2022). 10.1002/adma.202110140 35122340 10.1002/adma.202110140

[67] X. Hou, T.P. Pollard, X. He, L. Du, X. Ju et al., “Water-in-eutectogel” electrolytes for quasi-solid-state aqueous lithium-ion batteries. Adv. Energy Mater. **12**(23), 2200401 (2022). 10.1002/aenm.202200401

[68] J.-L. Yang, Z. Yu, J. Wu, J. Li, L. Chen et al., Hetero-polyionic hydrogels enable dendrites-free aqueous Zn-I_2_ batteries with fast kinetics. Adv. Mater. **35**(44), 2306531 (2023). 10.1002/adma.202306531 10.1002/adma.202306531 37608787

[69] Y. Xiong, H. Cheng, Y. Jiang, Z. Fan, X. Li et al., A novel water-reducer-based hydrogel electrolyte for robust and flexible Zn-I_2_ battery. Energy Storage Mater. **74**, 103981 (2025). 10.1016/j.ensm.2024.103981

[70] C. Li, T. Wang, H.C.J. Lai, S.W. Park, W.Y.K. Chan et al., Hydrophobic-unit-regulated hydrogel electrolytes with high water content and low salt concentration for high-voltage aqueous batteries. Joule **9**(4), 101827 (2025). 10.1016/j.joule.2025.101827

[71] M. Chen, J. Chen, W. Zhou, X. Han, Y. Yao et al., Realizing an all-round hydrogel electrolyte toward environmentally adaptive dendrite-free aqueous Zn-MnO_2_ batteries. Adv. Mater. **33**(9), e2007559 (2021). 10.1002/adma.202007559 33511697 10.1002/adma.202007559

[72] Y. Cheng, X. Chi, J. Yang, Y. Liu, Cost attractive hydrogel electrolyte for low temperature aqueous sodium ion batteries. J. Energy Storage **40**, 102701 (2021). 10.1016/j.est.2021.102701

[73] S. Farai Kuchena, Y. Wang, A full flexible NH_4_^+^ ion battery based on the concentrated hydrogel electrolyte for enhanced performance. Chemistry **27**(62), 15450–15459 (2021). 10.1002/chem.202102442 34331345 10.1002/chem.202102442

[74] K. Niu, J. Shi, L. Zhang, Y. Yue, M. Wang et al., A self-healing aqueous ammonium-ion micro batteries based on PVA-NH_4_Cl hydrogel electrolyte and MXene-integrated perylene anode. Nano Research Energy **3**(4), e9120127 (2024). 10.26599/nre.2024.9120127

[75] W. Lv, J. Liu, Z. Shen, X. Li, C. Xu, Novel approaches to aqueous zinc-ion batteries: challenges, strategies, and prospects. eScience **5**(6), 100410 (2025). 10.1016/j.esci.2025.100410

[76] K. Xie, P. Zhu, D. Han, B. Zhang, X. Wang et al., Decoding “dead Mn” in MnO_2_ deposition/dissolution chemistry for energetic aqueous batteries: a perspective. Energy Materials and Devices **3**(3), 9370071 (2025). 10.26599/emd.2025.9370071

[77] Y. Tang, J.-H. Li, C.-L. Xu, M. Liu, B. Xiao et al., Electrode/electrolyte interfacial engineering for aqueous Zn-ion batteries. Carbon Neutralization **2**(2), 186–212 (2023). 10.1002/cnl2.54

